# Fast growth conditions uncouple the final stages of chromosome segregation and cell division in *Escherichia coli*

**DOI:** 10.1371/journal.pgen.1006702

**Published:** 2017-03-30

**Authors:** Elisa Galli, Caroline Midonet, Evelyne Paly, François-Xavier Barre

**Affiliations:** Institute for Integrative Biology of the Cell (I2BC), Université Paris-Saclay, CEA, CNRS, Université Paris Sud, Gif sur Yvette, France; A*STAR, SINGAPORE

## Abstract

Homologous recombination between the circular chromosomes of bacteria can generate chromosome dimers. They are resolved by a recombination event at a specific site in the replication terminus of chromosomes, *dif*, by dedicated tyrosine recombinases. The reaction is under the control of a cell division protein, FtsK, which assembles into active DNA pumps at mid-cell during septum formation. Previous studies suggested that activation of Xer recombination at *dif* was restricted to chromosome dimers in *Escherichia coli* but not in *Vibrio cholerae*, suggesting that FtsK mainly acted on chromosome dimers in *E*. *coli* but frequently processed monomeric chromosomes in *V*. *cholerae*. However, recent microscopic studies suggested that *E*. *coli* FtsK served to release the MatP-mediated cohesion and/or cell division apparatus-interaction of sister copies of the *dif* region independently of chromosome dimer formation. Here, we show that these apparently paradoxical observations are not linked to any difference in the dimer resolution machineries of *E*. *coli* and *V*. *cholerae* but to differences in the timing of segregation of their chromosomes. *V*. *cholerae* harbours two circular chromosomes, chr1 and chr2. We found that whatever the growth conditions, sister copies of the *V*. *cholerae* chr1 *dif* region remain together at mid-cell until the onset of constriction, which permits their processing by FtsK and the activation of *dif*-recombination. Likewise, sister copies of the *dif* region of the *E*. *coli* chromosome only separate after the onset of constriction in slow growth conditions. However, under fast growth conditions the *dif* sites separate before constriction, which restricts XerCD-*dif* activity to resolving chromosome dimers.

## Author summary

DNA synthesis, chromosome segregation and cell division must be coordinated to ensure the stable inheritance of the genetic material during proliferation. In eukaryotes, this is achieved by their temporal separation and the existence of checkpoint mechanisms that delay certain steps until others are completed. In contrast, replication, segregation and cell division are interconnected in bacteria. For instance, studies in slowly growing *Escherichia coli* cells revealed that sister copies of the replication terminus of its chromosome are tethered together at the division site by the binding of a protein, MatP, which interacts with the cell division machinery, to be orderly segregated by a cell division protein, FtsK, which assembles into oriented DNA pumps at the time of constriction. Here, we show using both genetic and fluorescent video microscopy that it is not the case when *E*. *coli* cells undergo multiple rounds of replication.

## Introduction

DNA synthesis, chromosome segregation and cell division must be coordinated to ensure the stable inheritance of the genetic material during proliferation. In eukaryotes, this is achieved by coupling the assembly and activity of the cell division apparatus to the assembly and activity of the mitotic spindle, a subcellular structure that serves to separate chromosomes. Yeast and animal cells also evolved a checkpoint mechanism that delays cell scission when chromatin remains trapped in the division plane. No such checkpoint exists in bacteria. Instead, they rely on a highly conserved protein, FtsK, to transport any trapped DNA from one daughter cell compartment to another during septation [1].

FtsK is a bi-functional protein. It includes an integral domain at its amino-terminus (FtsK_N_), a low complexity ‘linker’ region that lacks any evolutionarily conserved feature (FtsK_L_) and a conserved RecA-type ATPase fold at its carboxyl-terminus (FtsK_C_) [2]. It was initially discovered because of its essential role in cell division in *Escherichia coli* [3]. However, only FtsK_N_ and FtsK_L_ are implicated in the cell division process [4]. FtsK_C_ serves to transport DNA between daughter cell compartments before final scission [5,6]. It assembles into hexamers on double stranded DNA at the initiation of septation and uses the energy from binding/hydrolysis of ATP to translocate on it [7–10]. *E*. *coli* harbours a single circular chromosome with a single origin of replication, *oriC*. A winged helix domain at the extreme carboxyl-terminus of FtsK, FtsKγ, binds to specific 8-bp polar DNA motifs, the KOPS, which orientates its loading [11–13]. KOPS are over-represented on the *E*. *coli* chromosome. They point from *oriC* toward a specific 28 bp site, *dif*, within the replication terminus of the chromosome, *ter*. As a result, FtsK_C_ motors are directed towards *dif* [12]. Daughter chromosomes segregate progressively as they are replicated. However, the delay between the time of replication and the time of separation of sister loci is variable [14]. In particular, it was observed that *ter* sister copies remained close together at mid-cell until the very end of the cell cycle in different growth conditions [14–17]. This is at least in part explained by the binding of MatP, a protein that interacts with the cell division machinery, to specific DNA motifs within *ter* [18,19]. Microscopic observations of the cellular arrangement of pairs of chromosome loci under slow growth conditions recently suggested that FtsK translocation served to release the MatP-mediated cohesion and/or cell division apparatus-interaction of *ter* sisters in a KOPS-oriented manner, placing it at the centre of the coordination between the *E*. *coli* replication/segregation and cell division cycles [20].

Prior to this observation, FtsK translocation was only considered as a safeguard against the formation of chromosome dimers [21]. Chromosome dimers are generated by homologous recombination events between chromatid sisters during or after replication. They physically impede the segregation of genetic information at cell division, which generates a substrate for FtsK translocation. They are resolved by the addition of a crossover at *dif* by a dedicated pair of chromosomally encoded tyrosine recombinases, XerC and XerD. Xer recombination-deficient *E*. *coli* strains were extensively characterised by microscopy [22,23], growth competition [23], direct measurements of recombination rates at *dif* using density label assays derived from the Meselson and Stahl experiment [24–26] and the excision of a DNA segment inserted between two *dif* sites in direct repetition (*dif*-cassette) at the *dif* locus [2,23,27]. These studies demonstrated that chromosome dimers were due to *recA*-dependent homologous recombination initiated by either the *recF* or *recB* pathways and independently of the role of *recA* in SOS. They also suggested that chromosome dimers formed at a generation rate of less than 20% whatever the growth conditions. However, it was observed that mutations decreasing the processivity of replication forks increased their formation in agreement with the multiple roles played by homologous recombination in replication fork progression [26,28,29]. Growth competition and *dif*-cassette excision experiments further indicated that *dif* only functioned within the *ter* region, at the zone of convergence of the KOPS motifs [12,23,30,31]. Finally, density label and *dif*-cassette excision assays showed that recombination at *dif* took place at a late stage of cell division, after the initiation of septum constriction [25,32].

FtsK plays two essential roles in chromosome dimer resolution. First, KOPS-oriented FtsK-dependent DNA transport ensures that the two *dif* sites of a chromosome dimer are brought together at mid-cell [2,12,33]. Second, FtsK activates the addition of a crossover by the Xer recombinases via a direct interaction between FtsKγ and XerD [6,34,35]. The roles played by FtsK in chromosome dimer resolution explains the spatial restriction of the activity of *dif* on the chromosome to the KOPS convergence zone [12,23,30,31] while the temporal restriction of Xer recombination at chromosomal *dif* sites suggests that the action of FtsK_C_ is delayed compared to its recruitment to the septum [25,32]. Correspondingly, ectopic production of FtsK_C_ was sufficient to activate *dif* recombination outside of the KOPS convergence zone, independently of cell division and of *recA* [2,27]. Taken together, these results suggested that FtsK normally only acted on chromosome dimers. The mild phenotype of FtsK translocation deficient mutants and their suppression by the inactivation of *recA* corroborated this hypothesis [33,36–38], in apparent contradiction with the general role of FtsK in the release of MatP-mediated cohesion and/or cell division apparatus-interaction of *ter* sisters [20].

The dimer resolution machinery is conserved in almost all bacteria [21]. This is notably the case in *Vibrio cholerae*, which harbours two distinct, non-homologous circular chromosomes, chr1 and chr2. The dimer resolution sites of chr1 and chr2, *dif1* and *dif2*, respectively, differ in sequence. However, we previously showed that *V*. *cholerae* FtsK controlled the addition of a crossover by *V*. *cholerae* XerC and XerD at both sites [39]. As in *E*. *coli*, *V*. *cholerae dif*-cassette excision was restricted to the KOPS convergence zone within chr1 and chr2 *ter* regions and only took place after the initiation of septum constriction [40]. However, *dif-*cassette excision on both *V*. *cholerae* chromosomes was independent of *recA* [40]. In addition, it was too high to be solely explained by the estimated rate of chromosome dimer formation at each generation [39,40]. This observation prompted us to re-visit how the *E*. *coli* and *V*. *cholerae* chromosomes are managed at the time of cell division using a combination of careful *dif*-cassette excision assays and newly available fluorescence microscopy techniques.

Here, we show that the *E*. *coli recA*-dependency and *V*. *cholerae recA-*independency of *dif*-cassette excision are not determined by differences in the dimer resolution machineries of the two bacteria but by differences in the timing of segregation of their chromosomes: whatever the growth conditions, *V*. *cholerae* chr1 *ter* sister copies remain together at mid-cell until the onset of constriction, which increases the chances for FtsK to activate recombination at *dif* independently of *recA*. Likewise, we show that in slow growth conditions, *E*. *coli ter* sister copies separate after the onset of constriction and *dif*-recombination is independent of *recA*. In contrast, our results suggest that MatP does not prevent *E*. *coli ter* sister copies from separating away from each other and from mid-cell before constriction in fast growth conditions. We show that separation of *ter* sisters is independent of FtsK, which explains why recombination at *dif* becomes dependent on the formation of chromosome dimers by homologous recombination during fast growth.

## Results

### The *recA*-dependency of recombination at *dif* is species specific

**Fig 1:**
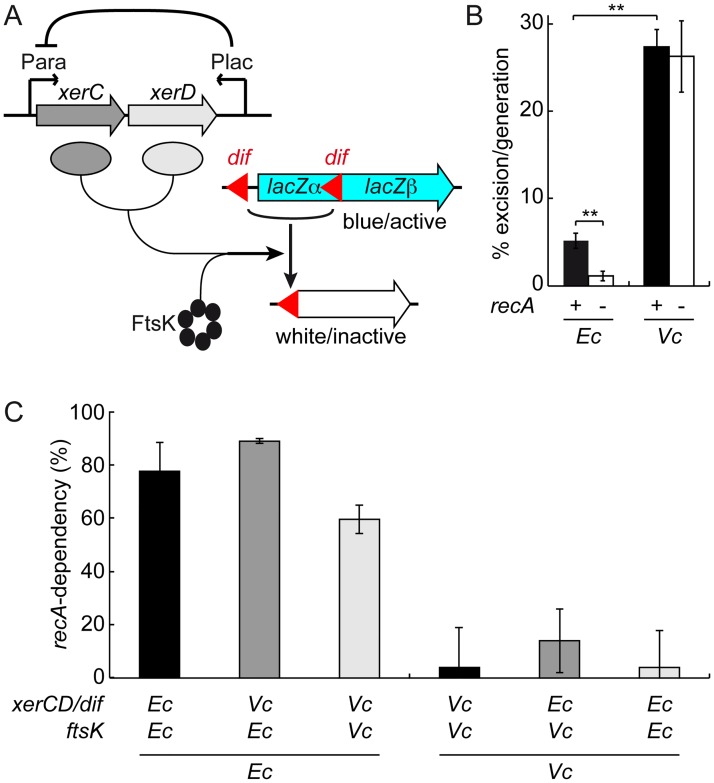
Genetic determinants of Xer recombination at *dif*. (A) Scheme of the *dif-*cassette excision genetic assay. (B) Influence of homologous recombination on the rate of *dif*-cassette excision in *E*. *coli* and *V*. *cholerae* cells grown in LB for 16 h and 3 h, respectively. Mean of 5 independent experiments. Ec: *E*. *coli*; Vc: *V*. *cholerae*; **: p<0.001 (t-test with a Two-tailed distribution). (C) *recA*-dependency of *dif-*cassette excision in *E*. *coli* and *V*. *cholerae* cells harbouring hybrid dimer resolution machineries. Mean of at least 3 independent experiments. Ec: *E*. *coli* cells grown for 16 h in LB; Vc: *V*. *cholerae* cells grown for 3 h in LB; *recA-*dependency: fraction of the *dif-*cassette excision rate that is linked to *recA*, 1-f_*recA-*_/f_*recA+*_.

We first checked whether the respective *recA*-dependency and *recA*-independency of *dif*-cassette excision in *E*. *coli* and *V*. *cholerae* was not due to differences in the design of the assays that were previously used in the two species. To this end, we created *dif-* and *dif1-*excision cassettes by introducing a first copy of these sites in the coding region of the *E*. *coli lacZ* gene in such a manner that the produced peptide retained its β-galactosidase activity and a second copy of the sites ahead of the *lacZ* ORF (Fig 1A). Recombination between the two sites of the cassettes excises a third of the *lacZ* ORF, which abolishes β-galactosidase production. Plating cells on X-Gal gives the cassette recombination frequency for different time points in growing cultures. We inserted the cassettes at the *dif* locus in strains in which the endogenous *lacZ*, *xerC* and *xerD* genes were deleted. XerC and XerD were produced from a *xerC*-*xerD* operon under the control of the arabinose promoter. The *E*. *coli lacZ* promoter and the *E*. *coli lacI* repressor gene were added in anti-orientation at the end of the operon to help repress any leaky expression of the recombinases (Fig 1A). In the case of *E*. *coli*, the *xerC-xerD* operon was introduced on a pBAD vector. Due to the instability of the vector in *V*. *cholerae*, the *xerC-xerD* operon was integrated in place of the *xerC* gene locus in the *V*. *cholerae* strains.

In a *dif*-excision cassette experiment, the frequency of recombination per cell per generation (f) is deduced from the initial and final Ratios of the non-recombined cells to the total number of cells (R_*i*_ and R_*f*_, respectively) and the number of divisions (n) that occurred during the course of the experiment with the following formula, f = 1-e^ln(R*f*/R*i*)/n^, which can be simplified into f = 1-e^ln(R*f*)/n^ because R_*i*_ normally equals 1. R_*f*_ is best monitored when in the 10–90% range. Large n minimises the possible error made on its estimation. Previous work showed that both conditions were reached with overnight cultures of *E*. *coli* strains in LB [23,27]. Using these conditions for our *lacZ dif*-cassette, we measured an excision rate in the order of 5% per generation (Fig 1B; n = 19.3±1.5, R_*f*_ = 36.4±5.4). The rate dropped to little more than 1% per generation in Δ*recA* cells (Fig 1B; n = 19.9±1.1, R_*f*_ = 79.8±7.3).

Previous results indicated that in the case of *V*. *cholerae* strains, only 3 h of growth in LB had to be used because of the elevated number of Xer recombination events at both *dif1* and *dif2* [40]. This short incubation time was large enough for n to reach a 6 to 7 value due to the fast growth rate of *V*. *cholerae*. Under these conditions, the excision rate of the *dif1*-cassette was in the order of 27% (Fig 1B; n = 6.8±0.5, R_*f*_ = 10±1.8) and was not affected by the deletion of *recA* (Fig 1B; in the order of 26%, n = 6.5±1.1, R_*f*_ = 13.7±2.5).

We confirmed that the respective *recA*-dependency and *recA*-independency of *dif*-cassette excision in *E*. *coli* and *dif1*-cassette excision in *V*. *cholerae* were not due to a difference in the number of divisions that cells performed during the course of the experiment by monitoring *E*. *coli dif*-cassette excision after only 8 h, i.e. at a time when n reached a 7 to 8 value (S1 Fig). Together, these results confirmed the species specificity of the *recA*-dependency of *dif* recombination.

### Dimer resolution machineries do not specify the *recA*-dependency of *dif* recombination

Cultures of *E*. *coli* and *V*. *cholerae recA*^-^ strains displayed similar loss of colony forming units (S2A Fig) and produced anucleate cells at a similar rate (S2B Fig), suggesting that the *E*. *coli recA*-dependence and *V*. *cholerae recA*-independence of *dif* recombination were not linked to any species specific role of RecA in the two bacterial species.

On the contrary, *V*. *cholerae* XerC and XerD seemed remarkably different from their *E*. *coli* counterparts in that they were known to act on sites with highly divergent central regions [39,41–43]. To check if the *recA*-independent cassette excisions observed in *V*. *cholerae* were linked to this peculiarity, we swapped the arabinose-inducible *xerC*-*xerD* operon and the excision cassettes of the *E*. *coli* and *V*. *cholerae* reporter strains. We also swapped the C-terminal domains of *E*. *coli* and *V*. *cholerae* FtsK to create reporter strains harbouring fully heterospecific Xer recombination systems. Cassette excision remained dependent on *recA* in the two *E*. *coli* hybrids and independent from it in the two *V*. *cholerae* hybrids, indicating that the FtsK/XerCD/*dif* systems did not specify the *recA*-dependency of *dif* recombination (Fig 1C).

### *E*. *coli* sister termini segregate ahead of septation in fast growth

As *E*. *coli* and *V*. *cholerae dif*-cassette excisions depend on the initiation of cell constriction [25,32,40] and are restricted to the *ter* regions [25,32,40], we wondered if differences in *recA*-dependency were linked to differences in the timing of segregation of the terminus of the *E*. *coli* and *V*. *cholerae* chromosomes with respect to the assembly of their cell division apparatus.

To test this hypothesis, we compared the position of *ydeV*, a locus 8 kb away from *dif* on the *E*. *coli* chromosome, and the position of the *V*. *cholerae* chr1 *dif1* locus in cells under exponential growth in liquid. LB-background fluorescence prevented the use of the exact same growth conditions as those of the cassette excision assays. However, *E*. *coli* and *V*. *cholerae dif*-cassette excisions remained *recA*-dependent and independent, respectively, in M9 supplemented with 10% of LB, 0.1% casamino acids and 0.2% of glucose (S3 Fig, M9-Rich). *V*. *cholerae* cells had a generation time of 23 min in this medium, which is only slightly longer than their 22 min LB generation time, whereas the generation time of *E*. *coli* cells increased from 24 min in LB to 40 min in M9-Rich.

**Fig 2:**
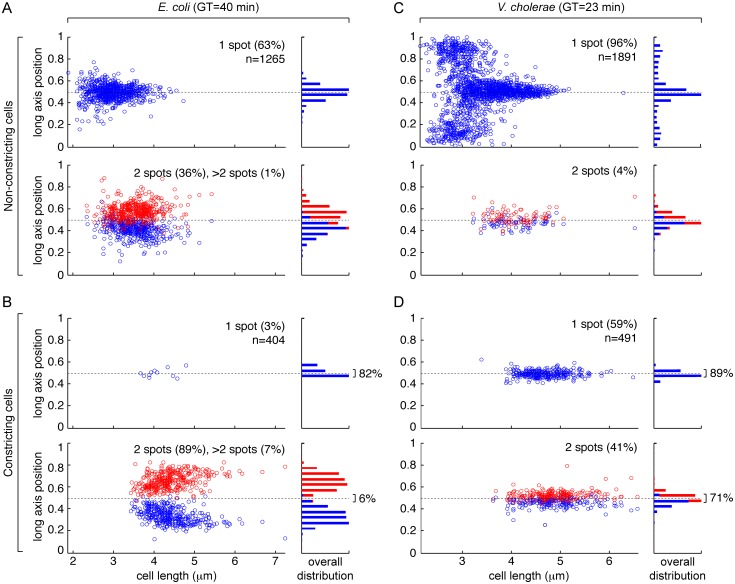
Fluorescence microscopy snapshot analysis of the position of the *dif* region in fast growing cells. (A) Position of the *ydeV* locus of the *E*. *coli* chromosome in cells with no visible indentation. (B) Position of the *ydeV* locus of the *E*. *coli* chromosome in cells with visible indentation. (C) Position of the *dif1* locus of *V*. *cholerae* chr1 in cells with no visible indentation. (D) Position of the *dif1* locus of *V*. *cholerae* chr1 in cells with visible indentation. GT: generation time; n: number of cells analysed; left panels: relative long axis position of foci as a function of cell length; right panels: overall distribution of foci positions; upper panels: cells presenting a single focus; lower panels: cells presenting 2 foci. Cells were arbitrarily oriented.

When grown in M9-Rich medium, 37% of *E*. *coli* cells displayed two or more *ydeV* sister loci before a constriction event could be detected (Fig 2A). The proportion of cells with two foci reached 89% when constriction was visible (Fig 2B, left panels). In addition, most of the foci were spatially separated with only 6% of them remaining close to mid-cell, i.e. at a distance from the cell centre of less than 5% of the cell length (Fig 2B, right panels). Thus, only ~8% of the foci, whether single or double, were in the immediate vicinity of the cell division apparatus in constricting *E*. *coli* cells with a 40 min generation time.

In contrast, a single *dif1* spot was observed in 96% of *V*. *cholerae* cells that were grown in M9-Rich medium and that presented no visible sign of constriction (Fig 2C). The spot was at mid-cell in the largest cells (Fig 2C). A single *dif1* spot was also observed in 59% of the cells in which constriction was visible (Fig 2D, upper left panel). 89% of them were in the immediate vicinity of the cell division apparatus at mid-cell, i.e. at a distance from the cell centre of less than 5% of the cell length (Fig 2D, upper right panel). 71% of the foci observed in the constricting cells with two spots (Fig 2D, lower left panel) were also in close vicinity of mid-cell (Fig 2D, lower right panel). Thus, 82% of the foci, whether single or double, were in the immediate vicinity of the cell division apparatus in constricting *V*. *cholerae* cells with a 23 min generation time.

Finally, we observed that in *V*. *cholerae* cells with a single *dif1* focus, the spot was located at a pole in the shortest cells, i.e. newborn cells (Fig 2C), whereas the *ydeV* focus of *E*. *coli* cells with a single spot was already positioned around mid-cell in the shortest cells (Fig 2A). Correspondingly, sister *dif1* spots remained located at mid-cell in the longest *V*. *cholerae* cells, i.e. ready to divide cells (Fig 2D), whereas sister *ydeV* spots relocated toward the 1/4 and 3/4 positions in the longest *E*. *coli* cells (Fig 2B).

These observations were consistent with the idea that sister *dif* sites split and migrated away from mid-cell before the onset of constriction could be manually detected. Sister *dif* sites separation would then prevent FtsK-mediated Xer recombination activation in *E*. *coli* unless a dimer was present. On the contrary, late sister *dif1* segregation would permit the action of FtsK on monomeric chromosomes in *V*. *cholerae*.

### Growth conditions influence sister *ter* segregation

The results of Fig 2 seemed to contradict the idea that FtsK drove the orderly segregation of *E*. *coli* sister *ter* independently of chromosome dimer formation [20]. However, the latter phenomenon had been documented for cells under extreme slow growth, with a generation time of 210 min [20]. It led us to suspect that growth conditions influenced sister *ter* segregation, with fast growth conditions accelerating their segregation.

**Fig 3:**
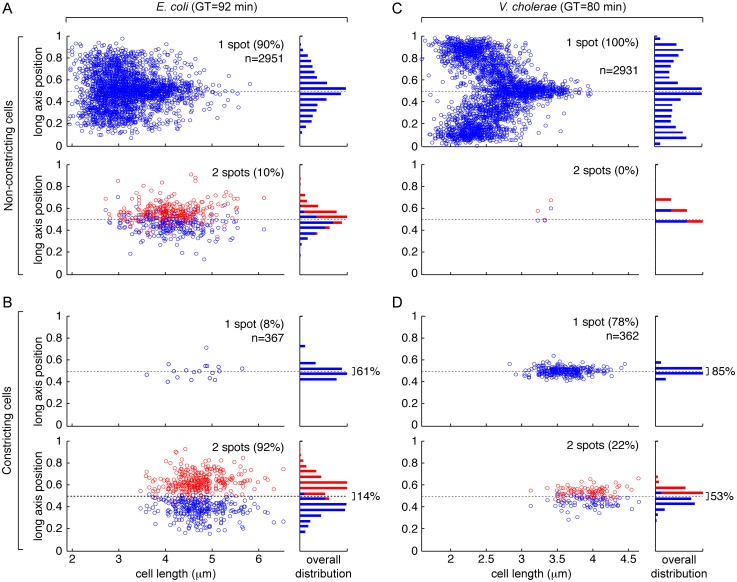
Fluorescence microscopy snapshot analysis of the position of the *dif* region in slow growing cells. (A) Position of the *ydeV* locus of the *E*. *coli* chromosome in cells with no visible indentation. (B) Position of the *ydeV* locus of the *E*. *coli* chromosome in cells with visible indentation. (C) Position of the *dif1* locus of *V*. *cholerae* chr1 in cells with no visible indentation. (D) Position of the *dif1* locus of *V*. *cholerae* chr1 in cells with visible indentation. GT: generation time; n: number of cells analysed; left panels: relative long axis position of foci as a function of cell length; right panels: overall distribution of foci positions; upper panels: cells presenting a single focus; lower panels: cells presenting 2 foci. Cells were arbitrarily oriented.

In order to verify this hypothesis, we inspected the localisation of the *ydeV* and *dif1* loci, in snapshot images of cells grown in liquid in minimal medium supplemented with only 0.2% of fructose (M9). Under this condition, *E*. *coli* cells presented a generation time of 92 min whereas *V*. *cholerae* cells had a generation time of 80 min. In the case of *E*. *coli*, 90% of the cells with no visible sign of constriction contained a single *ydeV* spot, the localisation of which reached mid-cell in the largest cells (Fig 3A). Most of the *E*. *coli* constricting cells (92%) still presented two *ydeV* spots (Fig 3B, lower left panel). However, 14% of them remained in close proximity to the cell centre, i.e. at a distance of less than 5% of the cell length (Fig 3B, lower right panel). Thus, foci, in the order of 18%, whether single or double, remained in the immediate vicinity of the cell division apparatus in constricting *E*. *coli* cells with a 92 min generation time. Correspondingly, the single *ydeV* spot of the shortest cells often located off the cell centre, toward the cell poles (Fig 3B).

A similar trend was observed for *V*. *cholerae* cells, with a single *dif1* spot detected in almost 100% of the cells that presented no visible sign of constriction (Fig 3C) and in 78% of the cells in which constriction was visible (Fig 3D). In total, 78% of the foci, whether single or double, were in the immediate vicinity of the cell division apparatus in constricting *V*. *cholerae* cells with an 80 min generation time, i.e. at a distance from the cell centre of less than 5% of the cell length.

These results suggested that growth conditions influenced sister *ter* segregation in both *E*. *coli* and *V*. *cholerae*.

### *E*. *coli dif*-cassette excision becomes *recA*-independent in slow growth conditions

**Fig 4:**
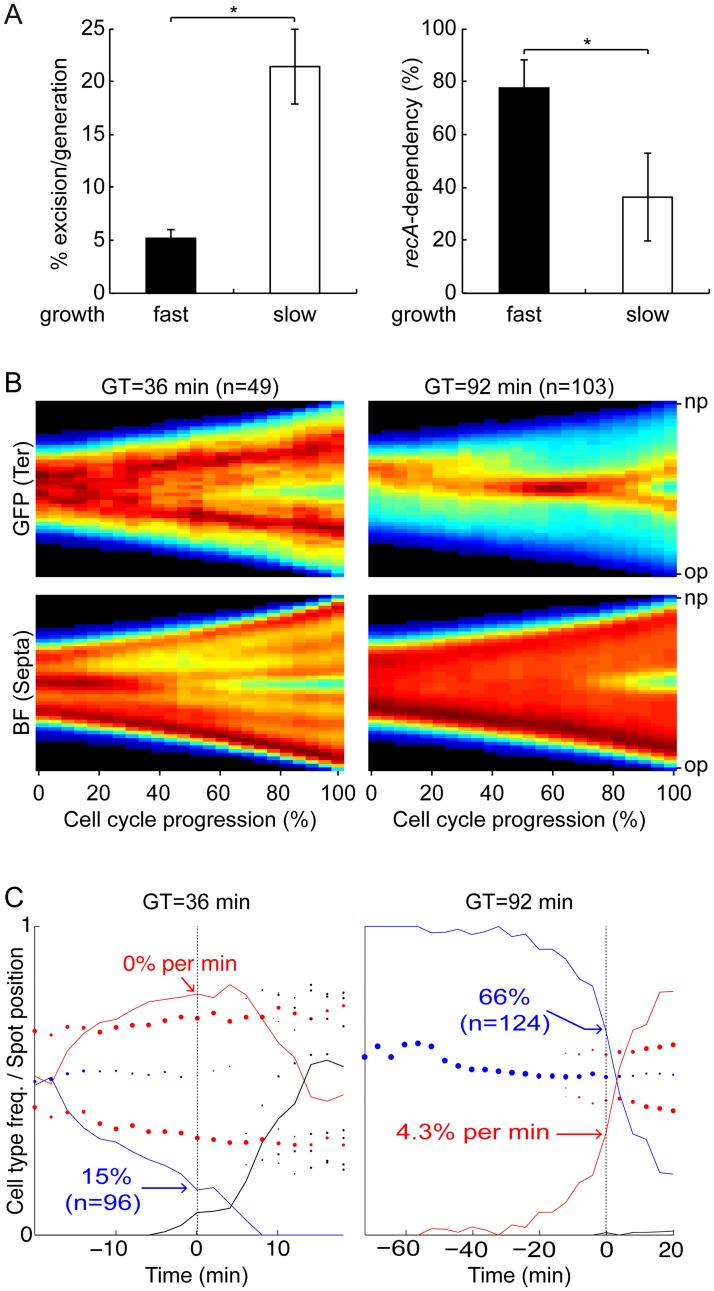
Initiation of multiple rounds of replication uncouples the final stages of chromosome segregation from cell division. (A) Rate of *dif*-cassette (left panel) and *recA*-dependency (right panel) of *dif-*cassette excision measured over 16 h in fast (LB) and slow (M9) growing *E*. *coli* cells. Mean of 5 independent experiments. *: p <0.01 (t-test with a Two-tailed distribution). (B) Consensus images summarising the cell cycle choreography of the *ydeV* fluorescence marker (upper panels, GFP) and the cell shape (lower panels, BF) in fast (left panels) and slow (right panels) growing *E*. *coli* cells. Only cells that were followed from birth to division were taken into consideration. At each time point, the maximal and minimal intensities of the projections were set to 1 and 0, respectively. We created images describing the evolution of the fluorescence and cell shape in the cell cycle by plotting the different projections of individual cells as a function of time using a jet colour code. In the heat maps, black corresponds to the lowest and dark red to the highest intensities. In the GFP maps the red areas indicate the presence of the *ydeV* marker, in the BF maps the green line indicates the Septa appearance. Individual fluorescence and cell shape images were compiled into consensus images summarising the results. Y-axis: position along the cell length. X-axis: cell cycle. GT: generation time; n: number of complete cell cycles that were analysed; op: old pole; np: new pole. 0: birth; 1: division. (C) Frequency of cells displaying a single spot (blue line), two spots (red line) and 3 or more spots (black line) as a function of the time before or after septation was detected. The Y-axis serves for both the cell type frequency lines and to the relative spot positions, with 0 indicating 0% frequency or the old pole position and 1 indicating 100% frequency or the new pole position. Left panels: M9-Rich; right panels: M9. The percentage of cells with a single *ydeV* spot at the time when septation was detected is indicated in blue with the number of observed cells (n) between parentheses. The percentage of new *ydeV* duplication events 0% per min and 4.3% per min represented at the time when septation was detected is indicated in red. GT: generation time.

We next wondered if the delay in the separation of the *E*. *coli ter* sisters that we observed in cells growing with a generation time of 92 min was sufficient to influence *dif*-cassette excision. Indeed, the rate of *dif*-cassette excision, as measured in a 16 h experiment, increased from 5% in LB to 20% in M9 (Fig 4A, left panel). In addition, a significant proportion of the observed recombination events (>60%) were now independent of *recA* (Fig 4A, right panel). These results were confirmed with 8 h *dif*-cassette recombination assays (S4 Fig).

### *E*. *coli ter* sister copies separate ahead of constriction in fast growth

As *dif*-cassette excisions depend on the initiation of cell constriction [25,32,40], the results of Fig 4A could be explained if separation of the *E*. *coli* chromosome *ter* sister copies was strictly connected to the onset of constriction under slow growth but that they separated ahead of constriction in fast growth, as suggested by the snapshot results of Figs 2 and 3. We therefore decided to follow the cell cycle choreography of sister *ydeV* loci by fluorescent video-microscopy to obtain direct evidence that their separation was strictly connected to the onset of constriction under slow growth but that it took ahead of it in fast growth.

In brief, a stack of 32 bright-field images below and above the focal plane of the cells and a single fluorescence image at the focal plane were taken at regular intervals. The bright-field stacks served to reconstruct a high definition image of the cell shapes. Cell genealogy was reconstructed, with cells arising from fully observed division events being oriented from their new pole (the pole originating from the division of the mother cell) to their old pole. Each of the individual cell lives were then analysed by two different methods.

First, we plotted the long cell axis projections of the fluorescence and reconstituted cell shape as a function of time for cells for which a complete cell cycle was recorded. At each time point, the maximal and minimal intensities of the projections were set to 1 and 0, respectively. We created images describing the evolution of the fluorescence and cell shape as a function of time by plotting the different projections of individual cells as a function of time using a jet colour code. Individual fluorescence and cell shape images were then compiled into consensus images summarising the results (Fig 4B). In the fluorescence consensus images (Fig 4B, upper panels, GFP), red colouring signals the position of the *ydeV* loci (*ter*). In the cell shape consensus images (Fig 4B, lower panels, BF), red colouring corresponds to regions of high bright-field signal with blue colouring at mid-cell signalling cell constriction (Septa). Use of the cell shape projections was as efficient as the use of a fluorescent derivative of the SPOR domain, which specifically labels nascent peptidoglycan at the septum, to detect constriction events (S5 Fig, [44]). Because projections were scaled from 0 to 1, each of the individual cell lives equally contributed to the consensus images. Consensus images indicated that in slowly growing cells, most sister termini separated and migrated away from mid-cell at the same time as constriction became visible, at approximately 80% of the cell cycle (Fig 4B, right panels). In addition, the *ydeV* fluorescence signals remained close to mid-cell up to the end of the cell cycle, and lagged at the new pole of newborn cells (Fig 4B), in agreement with the snapshot results of Fig 3. In contrast, in fast growing cells, sister termini already separated and located away from mid-cell at 40% of the cell cycle whereas constriction became visible only after 60% of the cell cycle had elapsed (Fig 4B, left panels). In addition, they already relocated to the 1/4 and 3/4 positions at the end of the cell cycle (Fig 4B).

Second, the position along the cell long axis of each fluorescence focus was manually determined as well as the position of any observed septation trace. This method could give a late estimate of the onset of constriction in cells. Note, however, that septation leads to the formation of a darker line across the cell section in our BF reconstructed images, which helps limit this bias (S6 Fig). We then aligned the cycles of cells for which the initiation of septation was monitored using as a reference the time when septation was first detected (Fig 4C, dashed vertical line at time 0). We computed the median positions of the fluorescence foci (Fig 4C, filled circles) and the frequency of cells with a single focus (Fig 4C, blue line and radius of blue circles), with two foci (Fig 4C, red line and radius of red circles) and with 3 or more foci (Fig 4C, black line and radius of black circles). It revealed that in fast growth conditions only 15% of *ter* sisters were not separated at the time when septation was first detected (Fig 4C, left panel, blue curve). Indeed, most *ter* sisters were already separated before the onset of constriction could be detected with our BF reconstruction method (Fig 4C, left panel, red curve), with the two foci having already moved away from mid-cell (Fig 4C, left panel, red spots). Finally, cells with 3 or 4 foci could be observed at late stages of the cell cycle, in agreement with the frequent birth of cells with duplicated *ter* sisters (Fig 4C, left panel, black spots and black line), which explained the decrease in the frequency of cells with two spots at these stages. In contrast, 100% of the slowly growing cells contained a single focus at birth and 66% of them still presented a single spot at the onset of constriction (Fig 4C, right panel, blue curve). In addition, the rate of duplication of sister termini was maximal at the onset of constriction, with 4.3% of new duplication events per min (Fig 4C, right panel, red curve).

Taken together, these results suggested that separation of sister copies of the *E*. *coli* chromosome *ter* region was connected to the onset of constriction under slow growth but that they separated ahead of constriction in fast growth.

It could somehow seem surprising that, in half of the cells grown in M9-Rich, 2 *ydeV* spots were visible at birth and 3–4 *ydeV* spots were visible at the time of division (Fig 4C) whereas the shortest cells of our snapshot image analysis results only had a single *ydeV* spot and the longest cells 2 *ydeV* spots (Fig 2). We cannot rule out the possibility that differences in the video-microscopy and snapshot image observations are due to differences in the growth condition of the cells in the two sets of experiments. Indeed, the median generation time of cells observed by video-microscopy was 10% shorter than the generation time of cells grown in liquid. However, we wish to emphasize that differences in the methods of analysis of snapshot images and time-lapse experiments are sufficient to explain the observed differences. Snapshot image analysis permits to observe a distorted cell cycle based on cell length instead of cell age because there is a considerable variation in the length of dividing and newborn cells, which is linked to the intrinsic randomness of growth and cell cycle regulation [16,45]. In addition, it is difficult to assess during the segmentation of snapshot images if joint cells correspond to (i) randomly juxtaposed cells, (ii) daughter cells from a recent cell scission event or (iii) the two halves of a cell in the process of constriction. It is essential to differentiate the first case from the two others, which implies the use of cell segmentation parameters that overestimate cell scission events, i.e. tend to separate the two halves of a cell in the process of constriction. As a result, some non-constricting cells shown in Fig 2 probably correspond to the two halves of a mother cell before scission. In contrast, time-lapse observations permit to unambiguously determine when cell scission has occurred because once separated the two daughter cells re-orientate and slide along each other. Correspondingly, in our video-microscopy experiments, the median length of cells just before cell scission was found to be in the order of 8 μm whereas the longest cell segments in snapshot images were in the order of 7 μm.

### MatP does not maintain *E*. *coli ter* sister copies at the division site in fast growth

**Fig 5:**
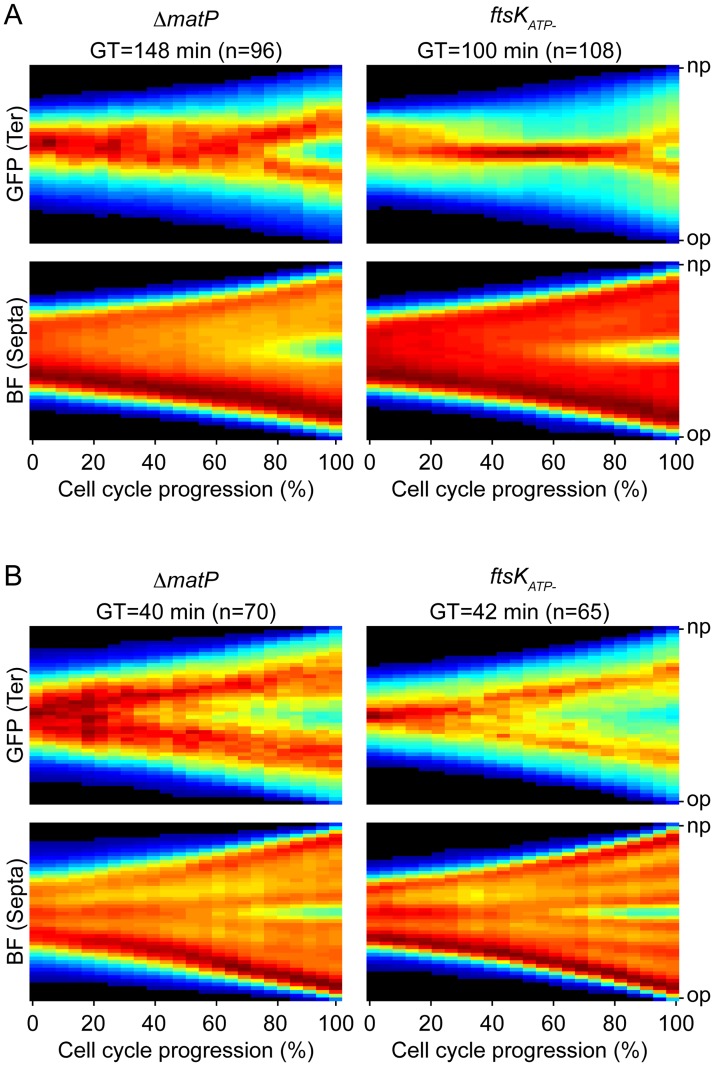
*ter* segregation in *matP*^-^ and *ftsK*_*ATP-*_ cells. (A) Slow growth conditions (M9). (B) Fast growth conditions (M9-Rich). Consensus images summarising the cell cycle choreography of the *ydeV* fluorescence marker (upper panels, GFP) and of the cell shape (lower panels, BF) in *matP*^-^ (left panels) and *ftsK*_*ATP-*_ (right panels) *E*. *coli* cells. GT: generation time; n: number of complete cell cycles analysed by fluorescence video-microscopy.

The *ter* domain of the *E*. *coli* chromosome harbours MatP-specific DNA binding motifs [19]. MatP interacts with ZapB, an early component of the cell division machinery [18]. As the MatP-ZapB interaction was shown to tether sister copies of *ter* loci at mid-cell and prevent their separation until the very end of cell division in cells grown on minimal media [18], we decided to check the action of MatP under our slow (M9) and fast (M9-Rich) growth conditions. When compared to the doubling time of *matP*^+^ *E*. *coli* cells, the generation time of *ΔmatP* cells increased by almost 60% in M9 and 10% in M9-Rich (Fig 5A and 5B, *ΔmatP*). Inspection of the cell contour and *ydeV* fluorescence consensus images further indicated that sister copies of the *ydeV* locus separated further ahead of the initiation of septation in the *ΔmatP* cells than they did in the *matP*^+^ cells: they separated at 70% of the cell cycle whereas constriction became visible at 80% of the cell cycle in M9 (Fig 5A, *ΔmatP*); most sister copies of the *ydeV* locus were already separated at 20% of the cell cycle whereas constriction was only visible at 60% of the cell cycle in M9-Rich (Fig 5B and S7 Fig). Taken together, these results indicated that MatP participated in chromosome organisation under both slow and fast growth conditions.

However, whether in rich or poor growth conditions, the number of *oriC* to *ter* foci remained constant during the growth of filaments induced by the addition of cephalexin (S8 Fig and S1 and S2 Movies). Cephalexin drives the formation of smooth filaments by blocking the activity of FtsI without disassembling the cell division apparatus and without impeding new rounds of chromosome replication [46]. These results suggested that MatP maintained *ter* regions attached to the division machinery for only a limited period of time between each new round of replication.

### FtsK translocation activity is not required for *E*. *coli ter* sister copies separation in fast growth

As FtsK translocation was shown to drive the orderly separation of sister copies of the terminus region of the *E*. *coli* chromosome under slow growth conditions [20], we next decided to check the role of FtsK in the separation of *ydeV* sister copies in M9 and M9-Rich conditions. To this end, we compared when *ydeV* sister copies separated with respect to the initiation of constriction in cells harbouring an ATPase deficient allele of *ftsK* (*ftsK*_*ATP-*_). We observed a similar 17% increase in the doubling time of *ftsK*_*ATP-*_ cells in M9 and M9-Rich conditions (Fig 5A and 5B, *ftsK*_*ATP-*_). In M9, sister copies of *ydeV* only separated at 90% of the cell cycle whereas constriction became visible at 70% of the cell cycle, thereby allowing the management of the terminus region by FtsK (Fig 5A, *ftsK*_*ATP-*_). However, sister copies of *ydeV* separated in cephalexin treated cells, suggesting that FtsK translocation is not absolutely required for sister *ter* separation under slow growth conditions (S8 Fig and S1 and S2 Movies). In M9-Rich, sister copies of *ydeV* separated at 40% of the cell cycle whereas constriction became visible at 70% of the cell cycle (Fig 5B, *ftsK*_*ATP-*_), demonstrating that *ydeV* sister copies separation did not require FtsK translocation in rich growth conditions. Careful inspection of individual cell lineages revealed frequent splitting of sister *ydeV* copies into 3–4 foci at the late stage of the cell cycle, further suggesting that FtsK translocation was not necessary to split the pairs of sister copies (S9 Fig). Indeed, *ydeV* sister copies seemed to separate 10% of the cell cycle time earlier in *ftsK*_*ATP-*_ cells than they did in FtsK^+^ cells in M9-Rich (Fig 5B, *ftsK*_*ATP-*_). The phenomenon was not unexpected since cells that require FtsK translocation to complete chromosome segregation during constriction (such as cells harbouring a chromosome dimer) are necessarily excluded from the analysis because they fail to complete their cell cycle.

## Discussion

### *dif*-cassette excision rates report the processing of chromosomes by FtsK

The activity of FtsK_C_ is the sole limiting factor for *dif*-recombination in WT cells, which suggested that *dif*-cassette excision could be used to monitor the processing of chromosomes by FtsK [2,6,39,40]. Indeed, restriction of the assembly of FtsK pumps to mid-cell at the onset of cell division [8] was reflected in the spatial and temporal restriction of *dif*-recombination to the *ter* region of chromosomes [2,23,40] and to the initiation of constriction [32,40]. Correspondingly, the low excision rate of *dif*-cassettes integrated at the *E*. *coli dif* locus and its dependence on *recA* were attributed to the low frequency with which the *ter* regions of monomeric chromosomes remained trapped in the septum at the onset of constriction [2,27]. However, this view was contradicted by recent microscopic observations, which suggested that FtsK promoted the orderly segregation of loci within the *E*. *coli ter* region, whether chromosome dimers were present or not [20], which raised the possibility that an as yet unknown mechanism restricted Xer recombination to chromosome dimers in *E*. *coli*.

The excision of *dif*-cassettes was not restricted to chromosome dimers in *V*. *cholerae* [40], further suggesting that the *E*. *coli recA*-dependence of *dif*-cassette excision might be due to a specific property of its dimer resolution system. The results we present demonstrate that it is not the case (Fig 1). Instead, they suggest that *recA*-dependency and *recA*-independency are due to the choreography of segregation adopted by the *ter* regions of chromosomes in the two species (Fig 2). In particular, the rate of *dif*-cassette excision increased and became less *recA*-dependent in conditions in which *E*. *coli ter* sister copies remained more frequently located at mid-cell at the onset of constriction (Figs 3 and 4). Taken together, these results suggest that differences in *dif*-cassette excision rates report the relative proximity of sister chromosomal regions at the time of constriction in fast and slow growing *E*. *coli* and *V*. *cholerae* cells.

### Fast growth conditions uncouple *ter* segregation and cell division in *E*. *coli*

Sister copies of the *ter* region of chromosomes co-localize at mid-cell until the initiation of cell division in both *E*. *coli* and *V*. *cholerae* [47,48], at least in part because of the MatP/*matS* macrodomain organisation system [18,19,40]. This mode of segregation participates in the coordination between chromosome segregation and cell division. In particular, a nucleoid occlusion factor, SlmA, impedes the assembly of the cell division machinery until a time when the only genomic DNA left at mid-cell consists of the sister copies of the terminus region of chromosomes [49,50].

Previous reports suggested that the separation of *ter* sisters was mediated by FtsK translocation, which stripped MatP off DNA during constriction [20,40]. It led to the idea that MatP and FtsK served to coordinate the final stages of chromosome segregation with cell division in bacteria [51]. Our observations of the position of sister termini in *V*. *cholerae* cells under slow and fast growth (Figs 3 and 2, respectively) and in *E*. *coli* cells under slow growth (Figs 3 and 4) are fully consistent with this idea: sister termini separated at a late stage of the cell cycle, concomitantly with cell constriction (Figs 2, 3 and 4); correspondingly, the chromosomal terminus region lagged at one pole (the new pole) of newborn cells (Figs 2, 3 and 4). In fast growth conditions, however, we found that the sister termini of the *E*. *coli* chromosome separated prior to the initiation of septation (Fig 4). They even often reached the 1/4 and 3/4 positions before the onset of constriction could be detected, emphasizing that *ter* segregation was independent from cell division (Figs 2 and 4). Together, these results suggested that *ter* segregation was generally independent of FtsK in fast growth conditions, which we confirmed by analysing the choreography of segregation of *ter* in *ftsK*_*ATP-*_ cells (Fig 5).

### MatP maintains *E*. *coli ter* sister copies at the division site for a limited amount of time

Our observations of Δ*matP* cells suggested that MatP still participated in the organisation of the *ter* region in both slow and fast growth conditions (Fig 5). What mechanism could explain FtsK-independent sister *ter* separation in fast growing *E*. *coli* cells (Fig 5 and S8 Fig)? We are attracted to the idea that new rounds of replication/segregation reorganise chromosomal DNA and in particular separate sister *ter* copies by pulling them towards opposite replication machineries. This model readily explains the differences between the action of FtsK in slow and fast growing cells and in cells treated with cephalexin: in slow growing cells, MatP holds sister copies of the terminus together at mid-cell after they are replicated. As the next round of replication/segregation is a long way off, separation of sister *ter* copies depends on FtsK translocation (Fig 4). Note, however, that FtsK translocation is not essential (Fig 5). In fast growing cells, overlapping rounds of replication/segregation break sister *ter* copies apart before the onset of constriction (Fig 4, [52]). The resulting 1/4 and 3/4 sister foci are separated or not depending on the advancement of the next/overlapping rounds of replication/segregation in each cell (Fig 4). In cephalexin treated cells new rounds of replication/segregation can likewise permit sister *ter* copies separation in the absence of constriction (S8 Fig). In contrast, overlapping replication rounds are absent and/or limited to one in *V*. *cholerae* cells under both slow and fast growth (Figs 2 and 3, [53]), which explains why FtsK always acts on sister *ter* regions (Fig 1). Future work will need to assess what are the different mechanisms that participate to the reorganisation of the bacterial nucleoid during growth and what is their relative contribution in different modes of growth [54].

## Materials and methods

### Plasmids and strains

Bacterial strains and plasmids used in this study are listed in S1 and S2 Tables, respectively. *V*. *cholerae* strains are derivatives of El Tor N16961 strain rendered competent by the insertion of *hapR* by specific transposition and constructed by natural transformation. *E*. *coli* strains are derivatives of MG1655, constructed by P1 transduction and/or integration/excision. Engineered strains were confirmed by PCR.

### Growth conditions

Growth media: LB (Luria-Bertani broth), M9-Rich (M9-MM supplemented with 0.2% glucose, 0.1% CAA, 10% LB and 1 μg/ml thiamine) and M9 (M9-MM supplemented with 0.2% fructose and 1 μg/ml thiamine). For the *in vivo* recombination assays, *V*. *cholerae* strains were grown in LB (generation time 22 min) and M9-Rich (generation time 23 min), and *E*. *coli* strains were grown in LB (generation time 24 min), M9-Rich (generation time 40 min) and M9 (generation time 92 min). For microscopy experiments *V*. *cholerae* strains were grown in M9-Rich and M9 (generation time 80 min), and *E*. *coli* strains were grown in M9-Rich and M9.

### *In vivo* recombination assay

*V*. *cholerae*: reporter cells were grown overnight in LB supplemented with 0.2 mM IPTG. Cultures were diluted in the morning in LB and grown at 37°C until they reached an OD_600_ comprised between 0.2 and 0.5. They were then diluted to an OD_600_ of 0.02 in LB supplemented with 0.1% L-Arabinose and grown at 37°C for 3 h. Serial dilutions of the cells were spread on LB agar plates supplemented with X-Gal (80 μg/ml) and IPTG (0.2 mM) before and after the induction of recombination.

*E*. *coli*: chemically competent reporter cells (rubidium chloride) were transformed with pCM165 or pCM166, inoculated in fresh media (LB, M9-Rich or M9) supplemented with 0.1% L-Arabinose and 100 μM ampicillin and grown at 37°C for 8 h or 16 h. Serial dilutions of the cells were spread on LB agar plates supplemented with ampicillin (100 μM), X-Gal (80 μg/ml) and IPTG (0.2 mM) before and after the induction of recombination.

Cells are not growing exponentially over the entire course of the experiments because (i) adaptation to the new growth conditions leads to a lag period at the beginning of the experiments and (ii) nutrient depletion leads to a stationary period at the end of the experiments. During both periods, cells do not divide. Therefore, recombination events can be almost entirely attributed to the intermediate exponential growth phase because *dif*-cassette excision takes place during cell division. The number of divisions (n) that took place during the exponential phase is deduced from the initial and final Number of cells in the cultures (N_*i*_ and N_*f*_, respectively) by the formula n = ln(N_*f*_/N_*i*_)/ln(2).

### Microscopy

For snapshot analyses, cells grown to exponential phase in M9 and M9-Rich were spread on 1% (w/v) M9 or M9-Rich agar pads, respectively (ultrapure agarose, Invitrogen). Phase contrast and fluorescence images were acquired using a DM6000-B (Leica) microscope. For time-lapse analyses, cells grown to exponential phase in M9 and M9-Rich were spread on a 1% (w/v) M9 or M9-Rich agar pads, respectively. Images were acquired using an Evolve 512 EMCCD camera (Roper Scientific) attached to an Axio Observe spinning disk (Zeiss). Pictures were taken every 2 min for cells grown in M9-Rich medium and every 4 min for cells grown in M9. At each time point, we took a stack of 32 bright-field images covering positions 1.6 μm below and above the focal plane. Cell contours were detected and cell genealogies were retraced with a MatLab-based script developed in the lab [49]. After the first division event, the new pole and old pole of cells could be unambiguously attributed based on the previous division events. A *lacO* array was inserted next to *dif1* and was detected using a LacI-mCherry fusion produced from the *lacZ* locus of *V*. *cholerae* chr1. A *parST1* motif was inserted at the *ydeV* locus and detected by production of a YGFP-ParBpMT1 from plasmid pFHC2973. A *lacO* array was inserted 15 kb from the *E*. *coli oriC* locus. For the joint detection of *oriC* and *ydeV* loci, LacI-mCherry and YGFP-ParBpMT1 fusions were produced from plasmid pAD16. Under these conditions, the tags did not interfere with the localisation of the loci [18,40,47]. The SPOR domain of FtsN was fused to mCherry and to the DsbA signal sequence to efficiently export it into the periplasm, the fusion was expressed from a P_BAD_ promoter induced with 0.01% L-Arabinose. SPOR localisation was inspected in M9. Cephalexin (10 μg/ml, final concentration) was added directly to the agarose slide. If not stated otherwise, leakiness of the promoter was sufficient for signal detection.

### Colony Forming Unit (CFU) assay

Wild Type and *recA*^-^ cells were grown to exponential phase, serial dilutions spread on plates and CFU calculated. Experiments were performed as triplicates of triplicates.

## Supporting information

S1 Table List of bacterial strains used in this study. (DOCX)

S2 Table List of plasmids used in this study. (DOCX)

S1 Fig Comparison of *dif*-cassette excision and *recA*-dependency in *E*. *coli* (Ec) and *V*. *cholerae* (Vc) with similar number of generations. Mean of at least 3 independent experiments. Error bars represent standard deviations. (A) Influence of homologous recombination on the rate of *dif*-cassette excision in *E*. *coli* and *V*. *cholerae* cells grown in LB for 8 h and 3 h, respectively. **: p<0.01; ****: p<0.0001; ns: p = 0.91 (One-way ANOVA with Tukey post-test). (B) *recA*-dependency of *dif-*cassette excision in *E*. *coli* under 16 h and 8 h of induction. ns: p = 0.42 (Unpaired two-tailed t test with Welch’s correction). Statistical analyses were performed using GraphPad Prism version 7.0b for Mac OS X, GraphPad Software, La Jolla California USA, www.graphpad.com. Error bars represent standard deviations. *recA-*dependency: fraction of the *dif-*cassette excision rate that is linked to *recA*, 1-f_*recA-*_/f_*recA+*_.
(TIF)

S2 Fig (A) Overnight *recA*^-^ and *recA*^*+*^ *E*. *coli* (Ec) and *V*. *cholerae* (Vc) cell cultures were diluted in fresh media and grown to an identical OD in the exponential phase. Colony forming units (CFU) of the cultures were determined by spreading serial dilutions on plates. In the graphs are shown the ratio of the CFU in *recA*^-^ over *recA*^+^ strains in Ec and Vc cells grown in LB, M9-Rich and M9. Experiments were performed as triplicates of triplicates. Error bars represent standard deviations. (B) Percentage of anucleate cells formed at each division in *recA*^-^ over *recA*^+^ strains of *E*. *coli* (Ec) and *V*. *cholerae* (Vc) grown in M9 and M9-Rich, as determined from 6 independent time-lapse experiments. Error bars represent standard deviations.
(TIF)

S3 Fig Rate of *dif*-cassette excision and *recA*-dependency in *E*. *coli* (Ec) and *V*. *cholerae* (Vc). Mean of at least 3 independent experiments. Error bars represent standard deviations. (A) Influence of homologous recombination on the rate of *dif*-cassette excision in *E*. *coli* cells grown in M9-Rich medium for 16 h. **: p<0.01 (Unpaired two-tailed t test). (B) *recA*-dependency of *dif-*cassette excision in *E*. *coli* cells grown in LB or M9-Rich. ns: 0.72 (Unpaired two-tailed t test). (C) Influence of homologous recombination on the rate of *dif*-cassette excision in *V*. *cholerae* cells grown in M9-Rich medium for 3 h. ns: 0.09 (Unpaired two-tailed t test). Mean of at least 3 independent experiments.
(TIFF)

S4 Fig Rate of *dif*-cassette excision (A) and *recA*-dependency (B) of *dif-*cassette excision measured over 8 h in fast (LB) and slow (M9) growing *E*. *coli* cells. Mean of at least 3 independent experiments. Error bars represent standard deviations. *: p <0.05 (with unpaired two-tailed t-test for (A) and with Welch’s correction for (B)).
(TIF)

S5 Fig (A) Consensus images of the cell shape (left panel) and SPOR domain (right panel) of *E*. *coli* cells grown in M9. (B) Cell shape (left panels) and SPOR domain (right panels) image choreographies of individual cells.
(TIFF)

S6 Fig (A) Time-lapse images of an *E*. *coli* cell grown in M9. The red arrow indicates the detection of constriction. (B) Mean pixel intensity along the cell length. Profile numbers correspond to the cell frame numbers of panel A. Profiles in which constriction could not be detected are shown in black. The profile in which constriction was first detected is shown in red.
(TIF)

S7 Fig Examples of individual cell cycles of *E*. *coli matP*^-^ cells growing in M9-Rich medium. In the left panels, representation of the manually detected *ydeV* spots and constriction sites. Green spots represent *ydeV* loci (fluorescent traces in right panels) and Black spots the constriction mark (bright field traces in central panels). For the fluorescent traces, at each time point, the maximal and minimal intensities of the fluorescence projections were set to 1 and 0, respectively. In the heat maps, black corresponds to the lowest and dark red to the highest intensities. In the GFP maps (right panels) the red lines indicate the presence of the *ydeV* spot, in the BF maps the green lines indicate the Septa appearance. Y-axis: 0, old cell pole; 1, new cell pole. X-axis: 0, 0% of the cell cycle; 1, 100% of the cell cycle.
(TIFF)

S8 Fig Time-lapse images of *ydeV* (ter) and *oriC* (ori) loci in *E*. *coli* cells grown in M9-Rich (A) or M9 (B) in the presence of 10 μg/ml cephalexin. NR: first frame in the time-lapse analysis in which new ori loci split. In the bottom right corner of each frame is indicated the time in minutes from the beginning of the time-lapse experiment.
(TIF)

S9 Fig Examples of individual cell cycles of *E*. *coli ftsK*_*ATP-*_ cells growing in M9-Rich medium. In the left panels, representation of the manually detected *ydeV* spots and constriction sites. Green spots represent *ydeV* loci (fluorescent traces in right panels) and Black spots the constriction mark (bright field traces in central panels). Y-axis: 0, old cell pole; 1, new cell pole. X-axis: 0, 0% of the cell cycle; 1, 100% of the cell cycle.
(EPS)

S1 Movie Time-lapse of *ydeV* (green) and *oriC* (red) loci localisation in *E*. *coli* cells. One frame was taken every 2 minutes. Cells were grown in M9-Rich. 10 μg/ml cephalexin was added to the agarose slide.
(AVI)

S2 Movie Time-lapse of *ydeV* (green) and *oriC* (red) loci localisation in *E*. *coli* cells. One frame was taken every 4 minutes. Cells were grown in M9. 10 μg/ml cephalexin was added to the agarose slide.
(AVI)
