# Supplementary material for: Fast growth conditions uncouple the final stages of chromosome segregation and cell division in Escherichia coli
Source: PLoS Genet. 2017 Mar 30;13(3):e1006702. doi: 10.1371/journal.pgen.1006702 (PMC5391129; doi:10.1371/journal.pgen.1006702)
Supplement: S1 Table — (DOCX) [file pgen.1006702.s001.docx]

**Table S1** List of strains used in this study

| ***E. coli*** |  |  |
| --- | --- | --- |
| **Strain** | **Genotype** | **Reference** |
| EG303 | MG1655, *ΔlacZ,* *xerD::aph* (KanR), *xerC::aacC1* (GmR), *Δdif:: dif1−lacZα−dif1-lacZβ* (1kb) | This study |
| EG304 | MG1655, *ΔlacZ,* *xerD::aph* (KanR), *xerC::aacC1* (GmR), *Δdif::* *dif_e.coli_−lacZα−dif_e.coli_-lacZβ* (1kb) | This study |
| EG315 | EG303, Δ*recA*::*cat* (CmR) | This study |
| EG316 | EG304, Δ*recA*::*cat* (CmR) | This study |
| EG375 | EG303, hybrid FsK with **e.coli**FtsK(NL)-**v.chol**FtsK(C) | This study |
| EG377 | EG375, *ΔrecA*::*cat* (CmR) | This study |
| EG457 | JJC7252, *hupA*::*mCherry-hupA-aph* (KnR) | This study |
| EG459 | EG457, Δ*recA-cat* (CmR) | This study |
| HU | *hupA*::*mCherry-hupA-aph* (KnR) | [1] |
| EG460 | ydeV, *lacO* array-*aph* (KnR) at *oriC* (15 Kb away) | This study |
| FX100 | *ftsK*^ATP-^ (K997A)*::cat* (CmR) | Lab collection |
| JJC7252 | ydeV, *araC::gfp-parBT1-pMT1* | Michel lab gift |
| recA | MG1655, *ΔrecA::cat* (CmR) | Lab collection |
| ydeV matP | ydeV, *ΔmatP::*FRT-*cat*-FRT (CmR) | This study |
| ydeV ftsK^ATP-^ | ydeV, *ftsK*^ATP-^(K997A)*::cat* (CmR) | This study |
| MG1655 |  | Lab collection |
| oriC | MG1655, *lacO* array-*aph* (KnR) at *oriC* (15 Kb away from midcell) | Lab collection |
| RM1 | MG1655, *ΔmatP::*FRT-*cat*-FRT (CmR) | [2] |
| ydeV | MG1655, *ydeV::parST1* | Boccard lab gift |
| ***V. cholerae*** |  |  |
| **Strain** | **Genotype** | **Reference** |
| ADV78 | EPV50, Δ*lacZ::*(P*_lac_::lacI-mCherry-parBT1-ygfp*) + LacOarray-*aph* (KnR) on chromosome 1 at position 1,56 Mb (1564103 bp) next to *dif1* + tet’-*parST1*-*cat*-’tet (CmR) inserted on chromosome 1 at position 0,05 Mb (53355 bp) | [3] |
| CMV42 | N16961, ChapR, *ΔlacZ,* Δ*xerD::*FRT-*sh ble* (ZeoR)-FRT Δ*xerC::*P_BAD_ **v.chol**XerCD, *Δdif1-*prophages*::dif1−lacZα−dif1-lacZβ*−*cat* (CmR) (1kb) | This study |
| CMV43 | N16961, ChapR, *ΔlacZ,* Δ*xerD::*FRT-*sh ble* (ZeoR*)*-FRT Δ*xerC::*P_BAD_ **e.coli**XerCD, Δ*dif1-*prophages*::dif1−lacZα−dif1-lacZβ*−*cat* (CmR) (1kb) | This study |
| CMV44 | CMV42, Δ*recA::aph* (KnR) | This study |
| CMV45 | CMV43, Δ*recA::aph* (KnR) | This study |
| EGV199 | EPV50, Δ*recA::aph* (KnR) | This study |
| EGV289 | CMV42, Δ*ftsKγ::aadA* (KnR) | This study |
| EGV291 | CMV43, Δ*ftsKγ::aadA* (SpecR) | This study |
| EGV458 | EPV231, Δ*recA::aph* (KnR) | This work |
| EPV50 | N16961 *ChapR* Δ*lacZ* (GmR) | [3] |
| EPV231 | EPV50, *ΔlacZ::*(P*_lac_::hupA-RFPT-FRT-sh ble*-FRT) (ZeoR) | This work |
| EPV379 | N16961, ChapR, Δ*lacZ*, Δ*xerD*, Δ*xerC::*P_BAD_ **v.chol**XerCD, Δ*dif1-prophages*:: *dif_e.coli_−lacZα−dif_e.coli_-lacZβ*−*cat* (CmR) (1kb) | This study |
| EPV382 | N16961, ChapR, Δ*lacZ*, Δ*xerD*, Δ*xerC::*P_BAD_ **e.coli**XerCD, Δ*dif1-prophages*:: *dif_e.coli_−lacZα−dif_e.coli_-lacZβ*−*cat* (CmR) (1kb) | This study |
| EPV383 | EPV379, Δ*recA::aph* (KnR) | This study |
| EPV385 | EPV382, Δ*recA::aph* (KnR) | This study |
| EPV392 | EPV379, hybrid FsK with **v.chol**FtsK(NL)-**e.coli**FtsK(C)-*arr2* (RifR) | This study |
| EPV394 | EPV382, hybrid FtsK with **v.chol**FtsK(NL)-**e.coli**FtsK(C)-*arr2* (RifR) | This study |
| EPV395 | EPV392, Δ*recA::aph* (KnR) | This study |
| EPV397 | EPV394, Δ*recA::aph* (KnR) | This study |
| EPV399 | CMV43, hybrid FtsKwith **v.chol**FtsK(NL)-**e.coli**FtsK(C)-*arr2* (RifR) | This study |
| EPV401 | CMV42, hybrid FtsK with **v.chol**FtsK(NL)-**e.coli**FtsK(C)-*arr2* (RifR) | This study |
| EPV403 | EPV399, Δ*recA::aph* (KnR) | This study |
| EPV406 | EPV401, Δ*recA::aph* (KnR) | This study |

**References**

1. Valens M, Thiel A, Boccard F. The MaoP/maoS Site-Specific System Organizes the Ori Region of the E. coli Chromosome into a Macrodomain. PLoS Genet. 2016;12: e1006309. doi:10.1371/journal.pgen.1006309

2. Mercier R, Petit M-A, Schbath S, Robin S, El Karoui M, Boccard F, et al. The MatP/matS site-specific system organizes the terminus region of the E. coli chromosome into a macrodomain. Cell. 2008;135: 475–485. doi:10.1016/j.cell.2008.08.031

3. David A, Demarre G, Muresan L, Paly E, Barre F-X, Possoz C. The two Cis-acting sites, parS1 and oriC1, contribute to the longitudinal organisation of Vibrio cholerae chromosome I. PLoS Genet. 2014;10: e1004448. doi:10.1371/journal.pgen.1004448
