# Supplementary material for: Fast growth conditions uncouple the final stages of chromosome segregation and cell division in Escherichia coli
Source: PLoS Genet. 2017 Mar 30;13(3):e1006702. doi: 10.1371/journal.pgen.1006702 (PMC5391129; doi:10.1371/journal.pgen.1006702)
Supplement: S2 Table — (DOCX) [file pgen.1006702.s002.docx]

**Table S2** List of plasmids used in this study

| **Name** | **Derived from** | **Properties** | **Source** |
| --- | --- | --- | --- |
| pCM54 | pUC18 | Vector of natural transformation carrying *SmR* (SpecR), *lacI*, *araC*, and P_BAD_ **v.chol**XerCD, flanking by UP and DWN regions of homologies around | [1] |
| pCM55 | pUC18 | Vector of natural transformation carrying *SmR* (SpecR), *lacI*, *araC*, and P_BAD_ **e.coli**XerCD, flanking by UP and DWN regions of homologies around | This study |
| pGD186 | pSW23T | Ori R6K, *cat*, *sacB*, partial *tetC* homologies to replace *dif1-*prophages*::dif1−lacZα−dif1-lacZβ*−*cat* (CmR) (1kb) in *V. cholerae* | Lab collection |
| pEP95 |  | Ori R6K, *cat*, *sacB*, partial *tetC* homologies to replace *dif1-*prophages*::dif_e.coli_−lacZα−dif_e.coli_-lacZβ*−*cat* (CmR) (1kb) in *V. cholerae* | This study |
| pEG292 | pUC18 | Vector of natural transformation carrying *bla* (AmpR), FRT-*she ble* (ZeoR)-FRT flanking by UP and DWN regions of homologies of *xerD* gene. To inactivate XerD in *V. cholerae* | This study |
| pMEV97 | pSW23T | Ori R6K, *cat* (CmR), *sacB*, *aph* (KnR) flanking with UP and DWN region of *recA* gene. To inactivate *recA* in *V. cholerae* | [2] |
| pAD23 | pUC18 | Vector of natural transformation carrying *bla* (AmpR),  Tet-*Sh ble* (ZeoR)-Tet cassette between UP and DW homologies in the regions of *dif1* and **without *dif1***. To delete *dif1* and put *tetC* homologies | [3] |
| pEG378 | pUC18 | Vector of natural transformation, *bla*, with cassette **v.chol**FtsK(NL)- **e.coli**FtsK(C)-*arr2* (RifR)-DWN **v.chol**FtsK. To have hybrids of FtsK in *V. cholerae* | This study |
| pFHC2973 | pUC18 | *bla*, *lacIq*, *lacO*-“cfp-parBP1”-“ygfp-parBpMT1”. Used for microscopy in *E. coli* to follow *ydeV* locus | [4] |
| pFX303 | pSC101 | *cat* (CmR), UP and DWN homologies around *ftsK.* Carrying ftsK100 K997A mutation. To have ATP- mutant of FtsK in *E. coli* | Lab collection |
| pFLP2 | pUC18 | *bla*, *sacB*, *flp* (Flippase). For FRT recombination to remove antibiotics resistance | Lab collection |
| pEG375 | pBR322 | *bla*, cassette parST1-FRT-*cat*-FRT flanking by VC1488 and *zapB* homologies. To delete *parS2* of chr1 | Lab collection |
| pCM165 | pSC101 | *bla, araC, lacIq*, P_BAD_ **e.coli**XerCD repressed by P*_lac_*. Expression of XerC in *E. coli* excision cassette assay | This study |
| pCM166 | pSC101 | *bla, araC, lacIq*, P_BAD_ **v.chol**XerCD repressed by P*_lac_*. Expression of XerCD in *E. coli* excision cassette assay | This study |
| pYB592 | pBAD33 | pACYC, *cat* (CmR), P_BAD_*::dsbA_ss_-mcherry-ftsN*[243-319] (*E. coli* SPOR domain, signal sequence from DsbA) | Yamaichi lab |
| pAD16 | pSC101 | pSC101, *bla* (AmpR), P*_lac_::lacI-mCherry-ygfp-parBpMT1* | Lab collection |

**References**

1. Midonet C, Das B, Paly E, Barre F-X. XerD-mediated FtsK-independent integration of TLCϕ into the Vibrio cholerae genome. Proc Natl Acad Sci. 2014;111: 16848–53. doi:10.1073/pnas.1404047111

2. Val M-E, Kennedy SP, El Karoui M, Bonne L, Chevalier F, Barre F-X. FtsK-dependent dimer resolution on multiple chromosomes in the pathogen Vibrio cholerae. PLoS Genet. 2008;4. doi:10.1371/journal.pgen.1000201

3. David A, Demarre G, Muresan L, Paly E, Barre F-X, Possoz C. The two Cis-acting sites, parS1 and oriC1, contribute to the longitudinal organisation of Vibrio cholerae chromosome I. PLoS Genet. 2014;10: e1004448. doi:10.1371/journal.pgen.1004448

4. Nielsen HJ, Ottesen JR, Youngren B, Austin SJ, Hansen FG. The Escherichia coli chromosome is organized with the left and right chromosome arms in separate cell halves. Mol Microbiol. 2006;62: 331–338. doi:10.1111/j.1365-2958.2006.05346.x
