# Supplementary figures and images for: Fast growth conditions uncouple the final stages of chromosome segregation and cell division in Escherichia coli
Source: PLoS Genet. 2017 Mar 30;13(3):e1006702. doi: 10.1371/journal.pgen.1006702 (PMC5391129; doi:10.1371/journal.pgen.1006702)

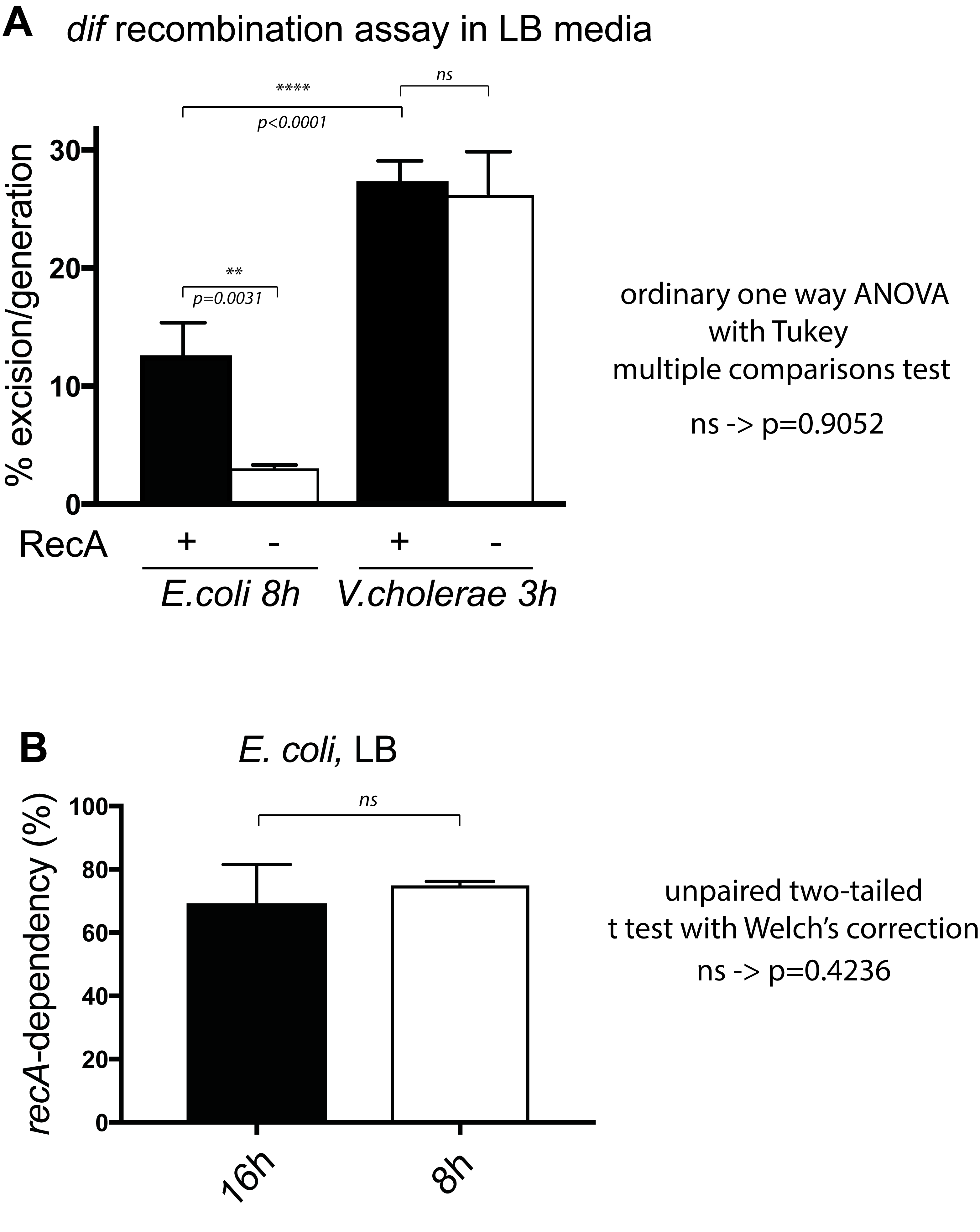

Supplement: S1 Fig — Mean of at least 3 independent experiments. Error bars represent standard deviations. (A) Influence of homologous recombination on the rate of dif-cassette excision in E. coli and V. cholerae cells grown in LB for 8 h and 3 h, respectively. **: p<0.01; ****: p<0.0001; ns: p = 0.91 (One-way ANOVA with Tukey post-test). (B) recA-dependency of dif-cassette excision in E. coli under 16 h and 8 h of induction. ns: p = 0.42 (Unpaired two-tailed t test with Welch’s correction). Statistical analyses were performed using GraphPad Prism version 7.0b for Mac OS X, GraphPad Software, La Jolla California USA, www.graphpad.com. Error bars represent standard deviations. recA-dependency: fraction of the dif-cassette excision rate that is linked to recA, 1-frecA-/frecA+. (TIF) [file pgen.1006702.s003.tif]

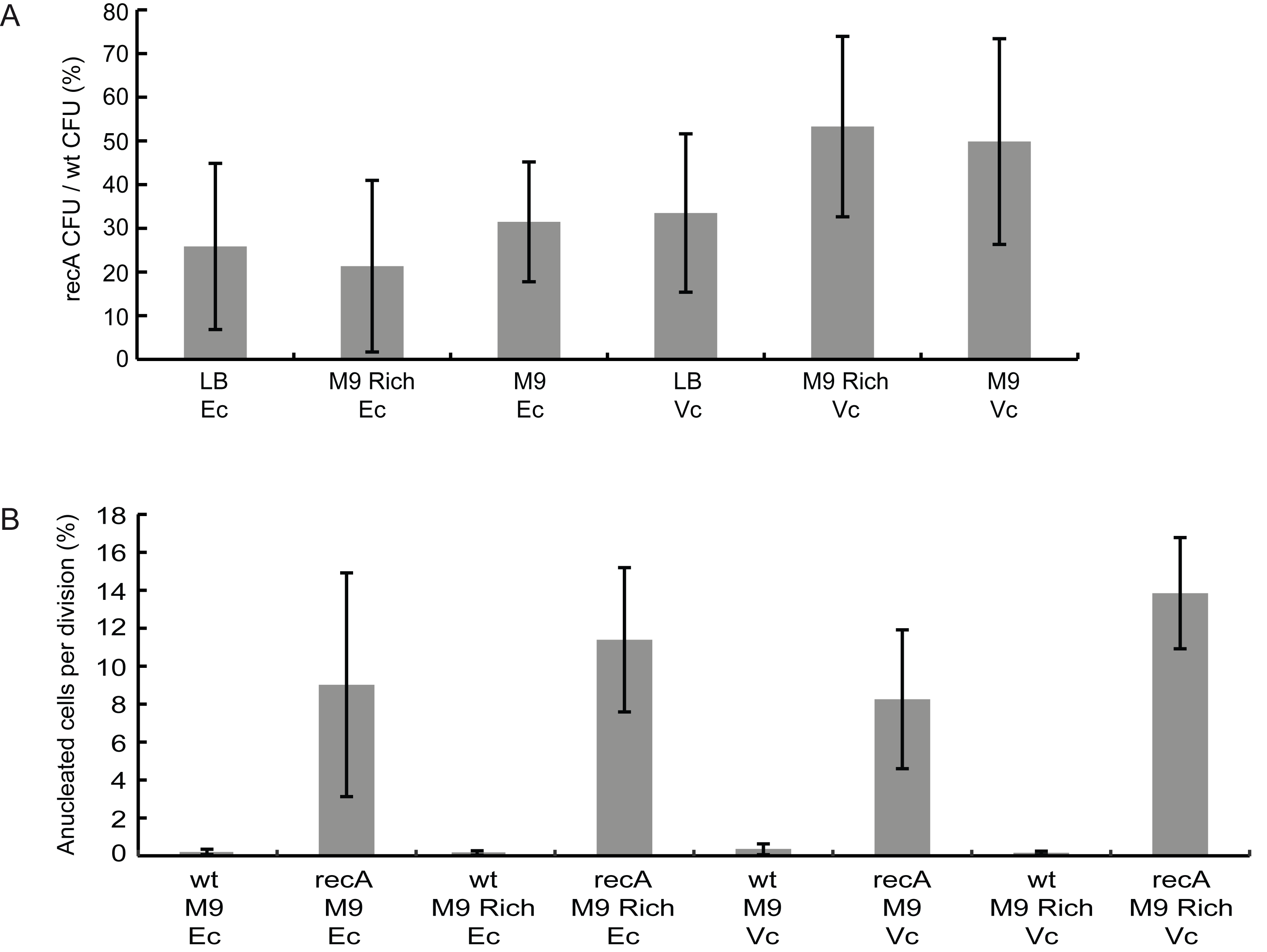

Supplement: S2 Fig — (A) Overnight recA- and recA+ E. coli (Ec) and V. cholerae (Vc) cell cultures were diluted in fresh media and grown to an identical OD in the exponential phase. Colony forming units (CFU) of the cultures were determined by spreading serial dilutions on plates. In the graphs are shown the ratio of the CFU in recA- over recA+ strains in Ec and Vc cells grown in LB, M9-Rich and M9. Experiments were performed as triplicates of triplicates. Error bars represent standard deviations. (B) Percentage of anucleate cells formed at each division in recA- over recA+ strains of E. coli (Ec) and V. cholerae (Vc) grown in M9 and M9-Rich, as determined from 6 independent time-lapse experiments. Error bars represent standard deviations. (TIF) [file pgen.1006702.s004.tif]

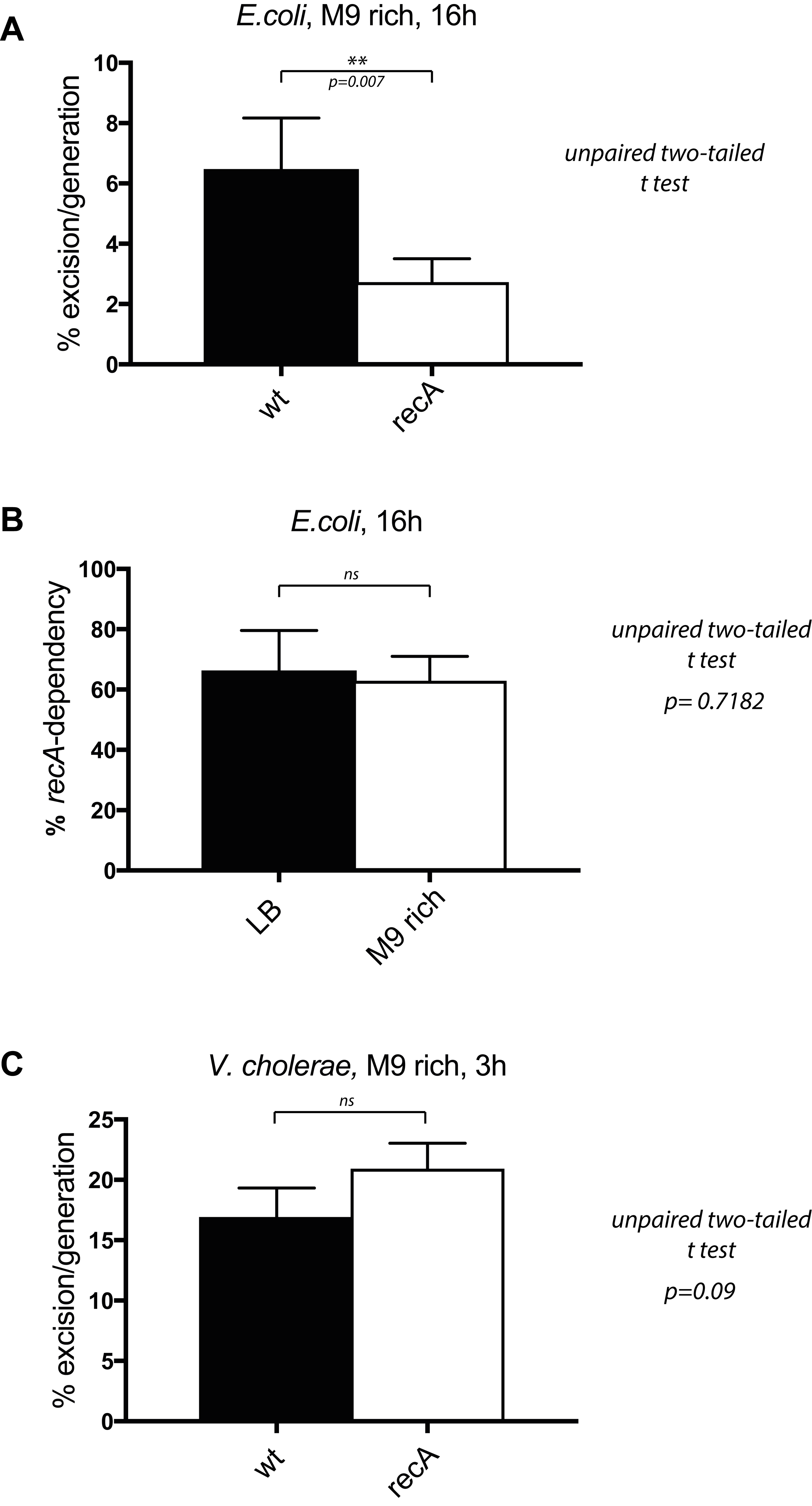

Supplement: S3 Fig — Mean of at least 3 independent experiments. Error bars represent standard deviations. (A) Influence of homologous recombination on the rate of dif-cassette excision in E. coli cells grown in M9-Rich medium for 16 h. **: p<0.01 (Unpaired two-tailed t test). (B) recA-dependency of dif-cassette excision in E. coli cells grown in LB or M9-Rich. ns: 0.72 (Unpaired two-tailed t test). (C) Influence of homologous recombination on the rate of dif-cassette excision in V. cholerae cells grown in M9-Rich medium for 3 h. ns: 0.09 (Unpaired two-tailed t test). Mean of at least 3 independent experiments. (TIFF) [file pgen.1006702.s005.tiff]

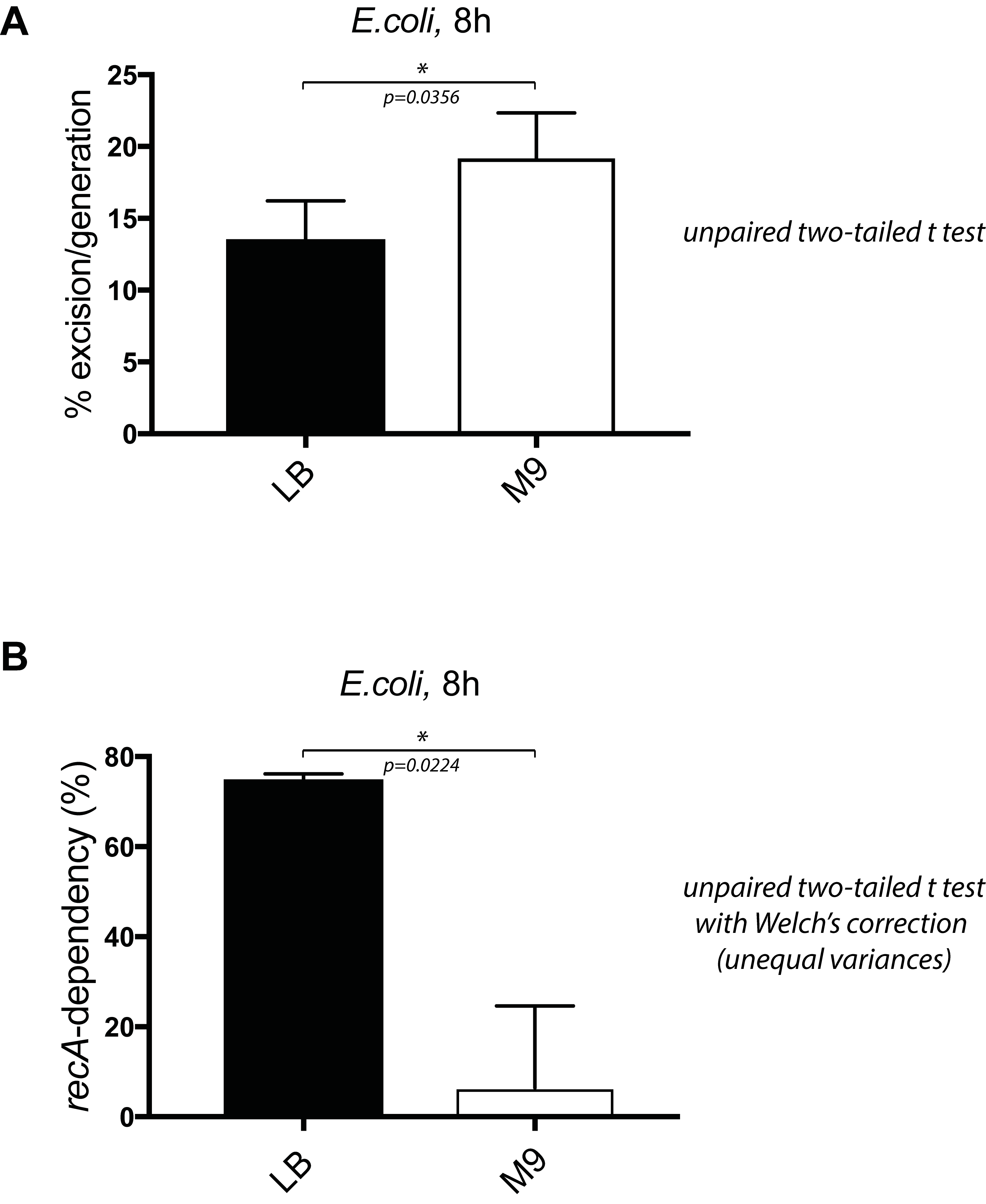

Supplement: S4 Fig — Mean of at least 3 independent experiments. Error bars represent standard deviations. *: p <0.05 (with unpaired two-tailed t-test for (A) and with Welch’s correction for (B)). (TIF) [file pgen.1006702.s006.tif]

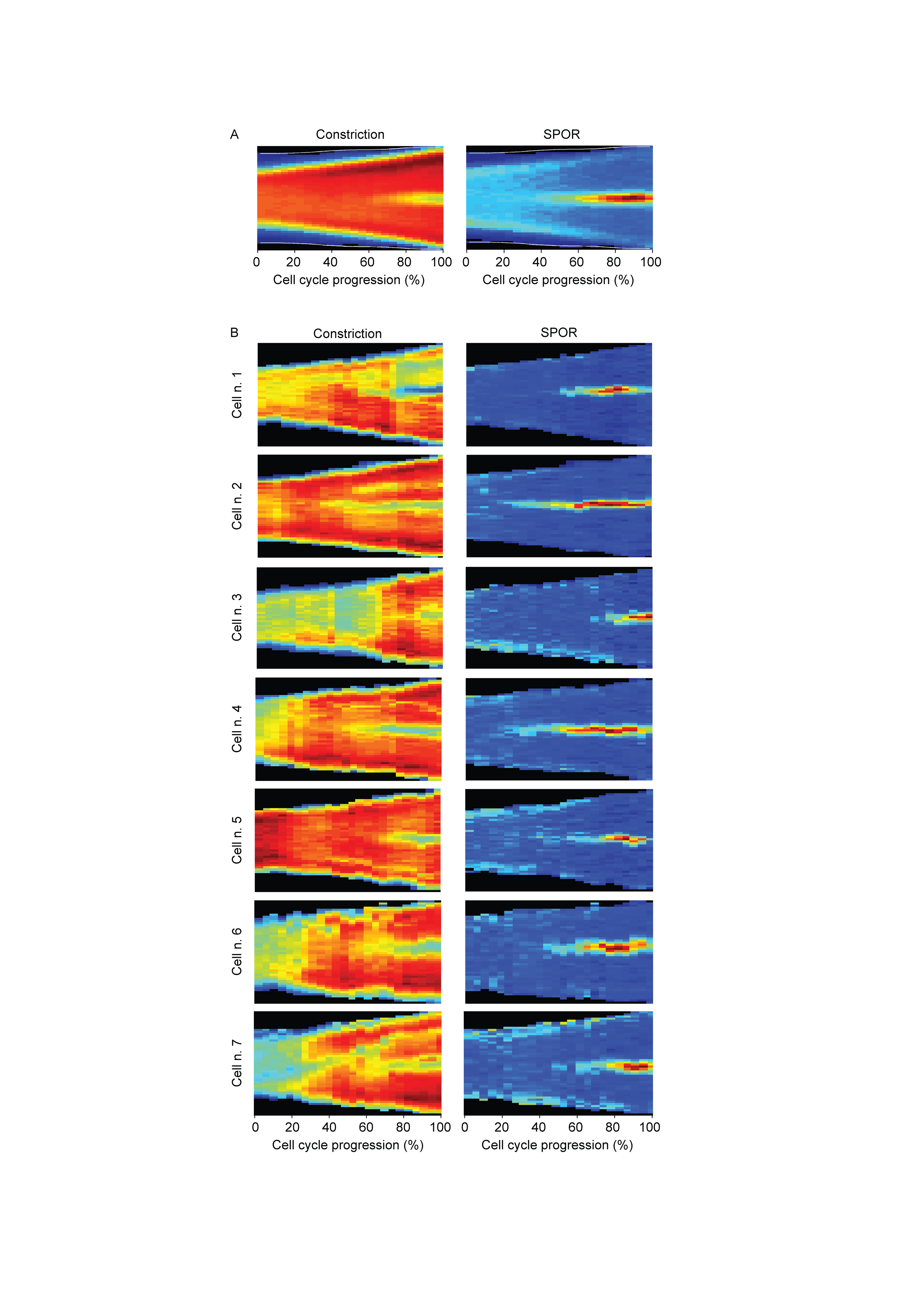

Supplement: S5 Fig — (A) Consensus images of the cell shape (left panel) and SPOR domain (right panel) of E. coli cells grown in M9. (B) Cell shape (left panels) and SPOR domain (right panels) image choreographies of individual cells. (TIFF) [file pgen.1006702.s007.tiff]

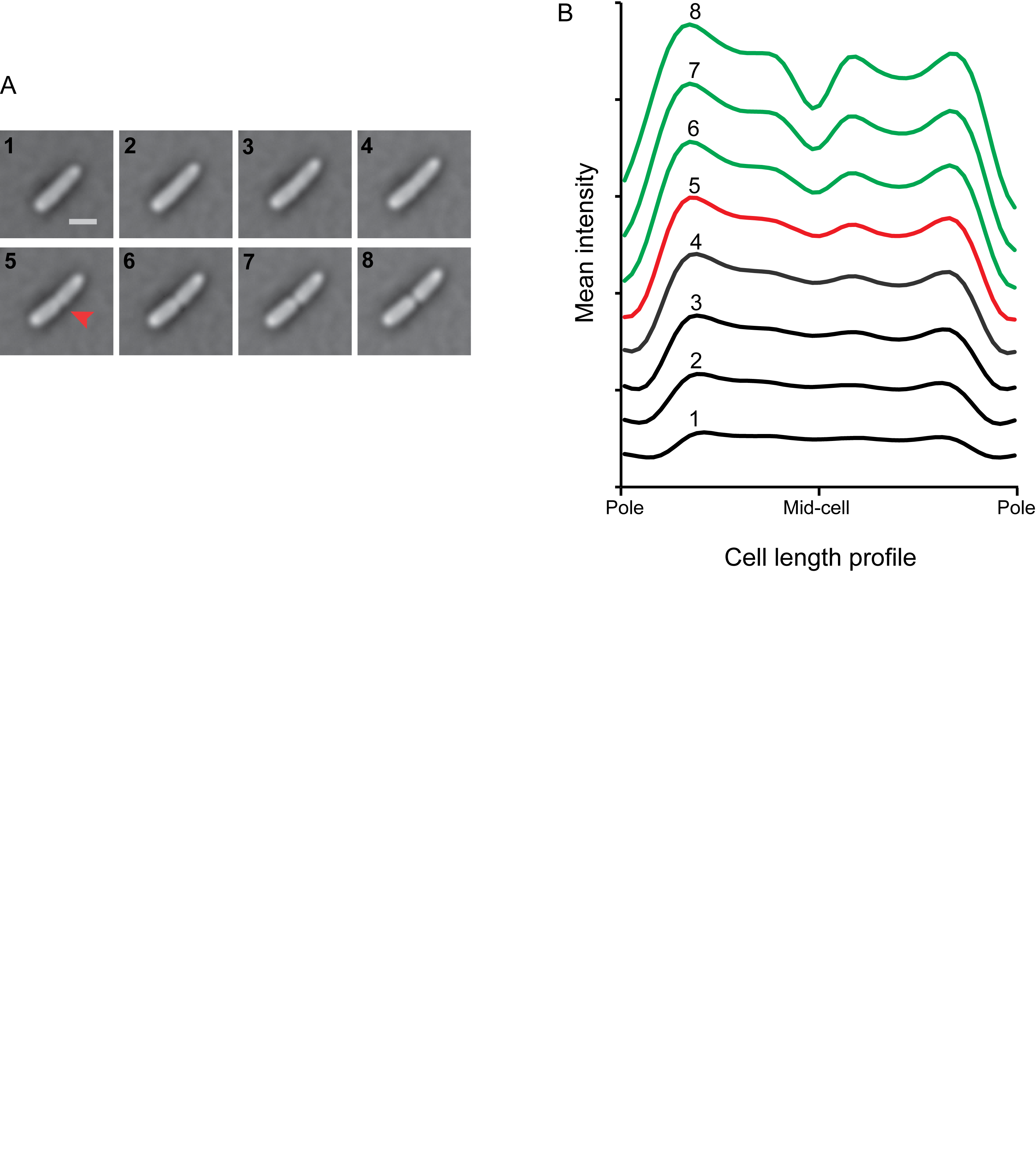

Supplement: S6 Fig — (A) Time-lapse images of an E. coli cell grown in M9. The red arrow indicates the detection of constriction. (B) Mean pixel intensity along the cell length. Profile numbers correspond to the cell frame numbers of panel A. Profiles in which constriction could not be detected are shown in black. The profile in which constriction was first detected is shown in red. (TIF) [file pgen.1006702.s008.tif]

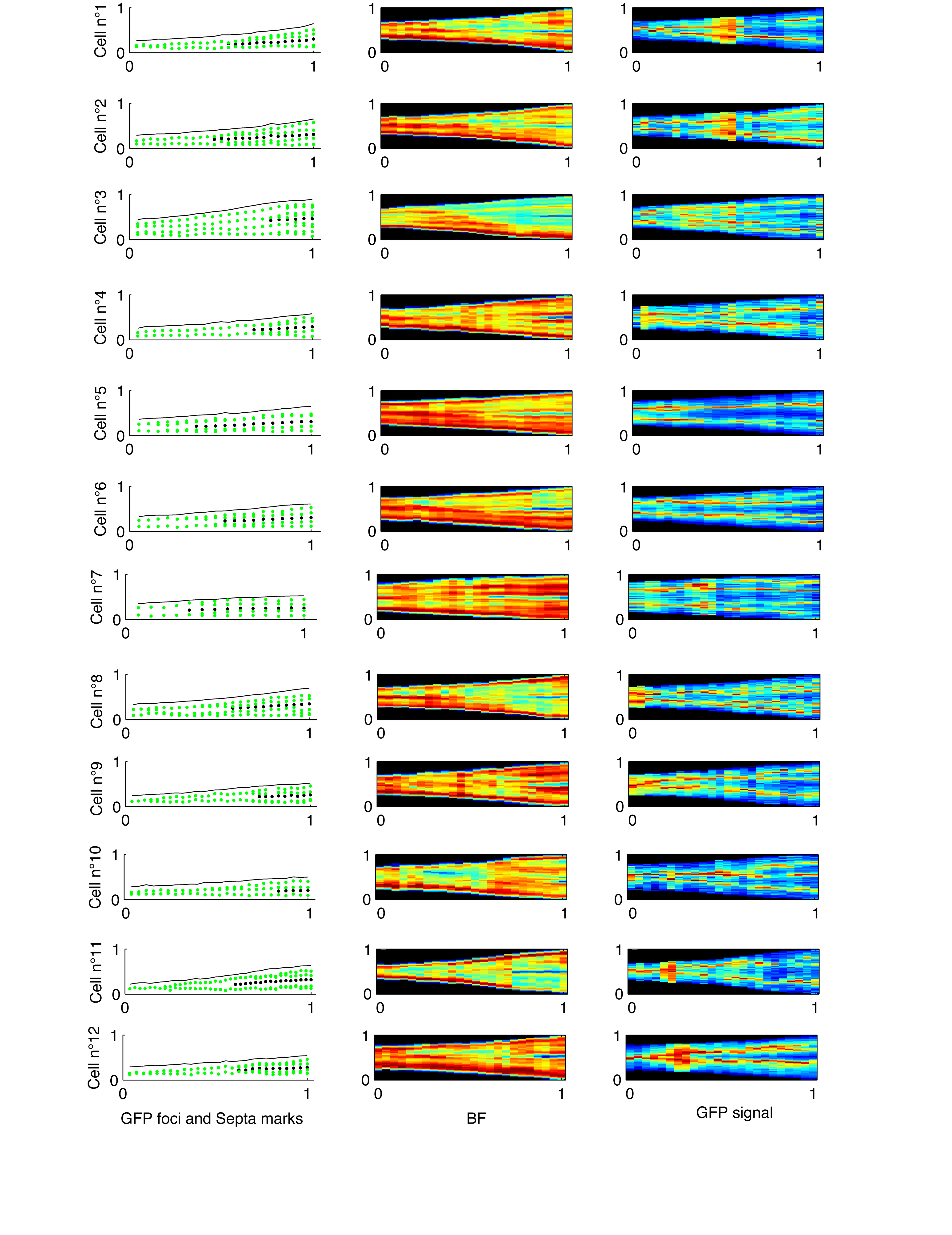

Supplement: S7 Fig — In the left panels, representation of the manually detected ydeV spots and constriction sites. Green spots represent ydeV loci (fluorescent traces in right panels) and Black spots the constriction mark (bright field traces in central panels). For the fluorescent traces, at each time point, the maximal and minimal intensities of the fluorescence projections were set to 1 and 0, respectively. In the heat maps, black corresponds to the lowest and dark red to the highest intensities. In the GFP maps (right panels) the red lines indicate the presence of the ydeV spot, in the BF maps the green lines indicate the Septa appearance. Y-axis: 0, old cell pole; 1, new cell pole. X-axis: 0, 0% of the cell cycle; 1, 100% of the cell cycle. (TIFF) [file pgen.1006702.s009.tiff]

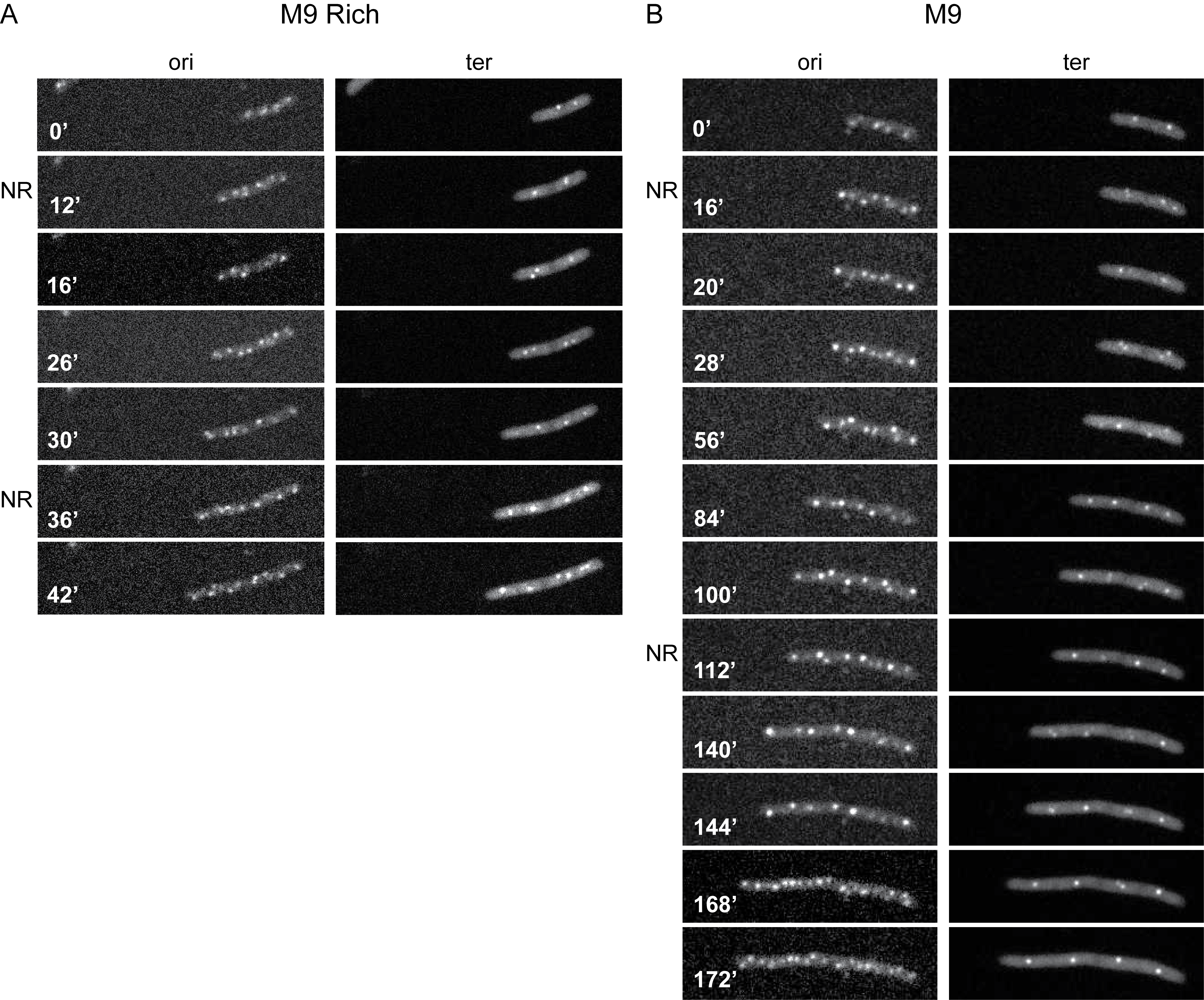

Supplement: S8 Fig — NR: first frame in the time-lapse analysis in which new ori loci split. In the bottom right corner of each frame is indicated the time in minutes from the beginning of the time-lapse experiment. (TIF) [file pgen.1006702.s010.tif]
